# Specificity Studies of the Venezuelan Equine Encephalitis Virus Non-Structural Protein 2 Protease Using Recombinant Fluorescent Substrates

**DOI:** 10.3390/ijms21207686

**Published:** 2020-10-16

**Authors:** Beáta Bozóki, János András Mótyán, Gyula Hoffka, David S. Waugh, József Tőzsér

**Affiliations:** 1 Department of Biochemistry and Molecular Biology, Faculty of Medicine, University of Debrecen, 4032 Debrecen, Hungary; bobea86@gmail.com (B.B.); hoffka.gyula@med.unideb.hu (G.H.); 2 Doctoral School of Molecular Cell and Immune Biology, University of Debrecen, 4032 Debrecen, Hungary; 3 MTA-DE Laboratory of Protein Dynamics, Department of Biochemistry and Molecular Biology, University of Debrecen, 4032 Debrecen, Hungary; 4 Macromolecular Crystallography Laboratory, Center for Cancer Research, National Cancer Institute at Frederick, Frederick, MD 21702, USA; waughd@nih.gov

**Keywords:** VEEV, Venezuelan equine encephalitis virus, non-structural protein, nsP2, protease, alphavirus, alphaviral protease, Group IV virus, specificity

## Abstract

The non-structural protein 2 (nsP2) of alphavirus Venezuelan equine encephalitis virus (VEEV) is a cysteine protease that is responsible for processing of the viral non-structural polyprotein and is an important drug target owing to the clinical relevance of VEEV. In this study we designed two recombinant VEEV nsP2 constructs to study the effects of an N-terminal extension on the protease activity and to investigate the specificity of the elongated enzyme in vitro. The N-terminal extension was found to have no substantial effect on the protease activity. The amino acid preferences of the VEEV nsP2 protease were investigated on substrates representing wild-type and P5, P4, P2, P1, P1′, and P2′ variants of Semliki forest virus nsP1/nsP2 cleavage site, using a His_6_-MBP-mEYFP recombinant substrate-based protease assay which has been adapted for a 96-well plate-based format. The structural basis of enzyme specificity was also investigated in silico by analyzing a modeled structure of VEEV nsP2 complexed with oligopeptide substrate. To our knowledge, in vitro screening of P1′ amino acid preferences of VEEV nsP2 protease remains undetermined to date, thus, our results may provide valuable information for studies and inhibitor design of different alphaviruses or other Group IV viruses.

## 1. Introduction

Venezuelan equine encephalitis virus (VEEV) is a New World alphavirus and belongs to the *Togaviridae* family of Group IV viruses. In the past few thousand years, the New World alphaviruses including Eastern equine encephalitis virus (EEEV) and Western equine encephalitis virus (WEEV) have evolved separately from those of the Old World including Sindbis virus (SINV), Semliki forest virus (SFV) and Chikungunya virus (CHIKV) [1]. VEEV is transferred to birds, horses, humans, and other vertebrates via arbovector (mosquitos), however, it is especially dangerous for equines, with an average fatality rate of 20–80% [2,3]. In humans, acute VEEV infections are usually defeated by innate and adaptive immune responses, however, about 14% of the infected humans develop neurological symptoms and about 1% of the infections result in lethal bugoencephalitis [3,4]. The inhaled virus can enter the brain via the olfactory neurons and as its viral particles are unusually resistant to desiccation and can be stably freeze-dried and aerosolized, therefore, VEEV has been regarded as potential biological weapon, that has been reportedly developed by several nations including the US and former Soviet Union [5,6].

Alphaviruses are small, spherical enveloped viruses and possess an ~11-kb-long ss(+)RNA genome [7]. The mRNA-like genome consists of two open reading frames (ORFs): (i) the first cistron codes for four non-structural proteins (nsPs) that are essential for viral replication complex formation and are translated in a form of a single polyprotein, nsP123 or nsP1234; while (ii) the second ORF encodes the structural proteins that form viral particles and are translated in the late stage of the infection.

The non-structural protein 2 (nsP2) is a central element of the VEEV life-cycle and has multiple enzyme activities. Its N-terminal region (Gly1-Ile456) is associated with ATP-ase and GTP-ase activity [8], RNA helicase activity [9], and RNA 5′-triphosphatase activity [10], while the C-terminal region (Met457–Cys794) controls 26S subgenomic RNA synthesis [11], downregulates minus-strand RNA synthesis late in infection [12,13], and directs nsP2 for nuclear transport [14]. Besides the listed activities, nsP2 is also essential in forming the alphavirus non-structural polyprotein replication complex via its proteolytic activity [15,16], as the C-terminal region of nsP2—frequently referred to as nsP2pro—consists of a papain-like cysteine protease domain linked to an S-adenosyl-L-methionine-dependent RNA methyltransferase domain (SAM MTase), for which the function has not been clarified. The active site of the protease (PR) is formed by a catalytic dyad including residues Cys477 and His546, while the substrate binding cleft is positioned between the protease and SAM MTase domains. Most of the residues that contribute to substrate binding are located in the protease domain; however, Hu et al. [5] have confirmed that at least three residues of the SAM MTase domain—including Arg662, Lys705 and Lys706—are involved in substrate recognition and in the regulation of the substrate-binding cleft structure. NsP2pro processes the non-structural polyprotein at three different sites located between nsP1/nsP2, nsP2/nsP3 and nsP3/nsP4 (referred to as nsP12, nsP23, and nsP34, respectively). All these sites share common sequential properties. According to the alignment of Strauss and Strauss [17] the residue in the P2 position is Gly in all three sites and the residue in P1 position is always an amino acid with a small side chain. Furthermore, the P3 site is considered as highly conserved owing to the relatively low number of the residues occurring therein. In contrast, P4 residues show greater variability suggesting that the major recognition signal for cleavage is likely to be displayed by residues P3-P2-P1. The amino acid sequence of P1′–P4′ positions are highly similar only within the same type of cleavage sites and differ significantly across them. This indicates that these residues—corresponding to the N terminus of the newly formed nsPs—are likely to be determined by their function in the released nsPs rather than by a requirement for cleavage site recognition.

According to Russo et al. [18], S1 pocket is formed by Asn475, Val476, and Ala509 residues that are thought to be either highly conserved or contain similar residues in most sequences (Figure S1), while backbone amides of Val476 and Cys477 likely form the oxyanion hole. The S2 subsite is mainly defined by the highly conserved Trp547 following the catalytic His546 and, together with the fact that the corresponding P2 site contains a highly conserved Gly, this structure of VEEV nsP2pro highly resembles the so-called “glycine specificity motif” of other cysteine proteases [19]. The S3 binding pocket is located on the SAM MTase domain, is composed of Ile698 and Met702, and is flanked by highly conserved Ala509 and His510 residues.

The characterization of nsP2pro in vivo and in vitro enzyme activity and specificity has been performed mainly in the context of SINV and SFV, and most of the previously performed mutagenesis studies were focused on activity of SIN and SFV nsP2pro on their cognate substrates mutated at P5-P1′ [20]. In the case of VEEV nsP2pro, only limited data are available regarding its specificity, although, the crystal structure of both the free and the E-64d-bound form has been solved [5,18]. Previously we have tested activities of three alphavirus proteases to investigate whether these enzymes are useful tools for affinity tag removal, similar to potyviral proteases including tobacco etch virus protease (TEV PR) that is already widely utilized for such purposes [20]. The activity of the recombinant SIN, SFV, and VEEV nsP2pro domains was investigated on different fusion proteins and on artificial oligopeptide substrates designed based on the P6-P6′ residues of their natural recognition sites. As the recognition sites of the three enzymes are highly similar, besides investigating the activity of the proteases on their cognate substrates, their cross-reactivity was also tested [20].

**Figure 1:**
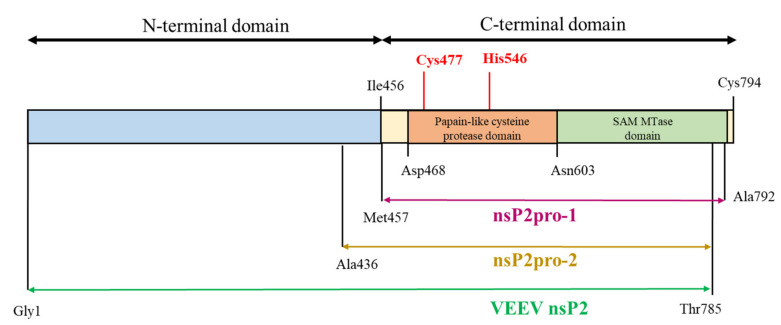
Schematic representation of the full-length Venezuelan equine encephalitis virus (VEEV) non-structural protein 2 (nsP2) and the corresponding enzyme construct. VEEV nsP2pro-1 (purple) is the construct applied in the previous studies [5,20]. Two different forms have been generated for this present study, in which nsP2pro-1 is completed with (i) the whole N-terminal domain of nsP2 (VEEV nsP2, olive) and (ii) with residues 436–457 (VEEV nsP2pro-2, yellow). Catalytic residues of the protease are shown by red color.

Kinetic parameters were determined previously on oligopeptide substrates for three enzyme–substrate pairs including SFV protease on its cognate SFV nsP12 and nsP34 cleavage site and VEEV protease on SFV nsP12. In that study [20], the VEEV protease was constructed based on Met457–Ala792, further referred to as nsP2pro-1, corresponding to the C-terminal domain of the nearly full-length nsP2 that lacks 793–794 terminal residues (Figure 1). The observed k_cat_/K_M_ values were substantially lower than those of potyviral proteases on their cognate substrates, furthermore, their ability to process fusion protein substrates was also relatively poor. We concluded that despite the fact that alphavirus proteases theoretically exhibit sufficient sequence specificity, in practice their cleavage properties in their investigated form could not offer any advantage over the already successfully utilized potyviral proteases.

Besides the biotechnological considerations, VEEV nsP2pro is also an antiviral drug target owing to (i) its essential function in the viral life-cycle as discussed above, and (ii) its potential central role in antagonizing the interferon response by cleaving host proteins corresponding to the generation of the innate immune response via short stretches of homologous host–pathogen protein sequences (SSHHPS) [21]. Mechanisms that antagonize early innate immune responses by proteolytic activity of viral nsPs are believed to have important function in other Group IV viruses, e.g., *Picornaviridae*, *Flaviviridae,* and *Coronaviridae.* Coronaviruses are under “super-intensive” investigation because several new pathogens contributing to recent global pandemics have emerged from these families including Middle East respiratory syndrome- and severe acute respiratory syndrome-associated coronavirus (MERS- and SARS-CoV), and SARS-CoV-2. In accordance with the similar mode of action of *Coronaviridae* and *Togaviridae*, structural similarities have been suggested by Compton et al. [22] between VEEV nsP2 and coronaviral papain-like proteases/deubiquitinases of the SARS- and MERS-CoV.

Currently there are no therapeutics approved for the treatment of VEEV infections, therefore, the characterization of the nsP2 proteolytic activity may be important for the development of successful inhibitor candidate molecules. In order to investigate the in vitro proteolytic activity of VEEV nsP2pro, we aimed to design two recombinant VEEV nsP2 constructs and study the effects of an N-terminal sequence elongation of the protease domain. In these new constructs, the sequence of nsP2pro-1 was expanded with (i) the whole N-terminal domain, further referred to as VEEV nsP2 or (ii) with the Ala436-Met457 region of VEEV nsP2, referred as nsP2pro-2 (see Figure 1). To study catalytic activity and specificity in vitro, we utilized such His_6_-MBP-mEYFP fusion protein substrates (His_6_, hexahistidine; MBP, maltose binding protein; mEYFP, monomeric enhanced yellow fluorescent protein). The recombinant proteins comprised a wild-type or a modified SFV nsP12 cleavage site sequence and were used as substrates in an Ni-NTA magnetic bead-based protease assay which has been developed in our laboratory [23,24,25].

## 2. Results

### 2.1. Expression Vectors Coding for VEEV nsP2 and nsP2pro-2

The VEEV nsP2 (1–785) was generated based on the assumption that the activity of CHIKV nsP2 was supposed to be dependent on the contributions of both N- and C-termini due to a possible interaction or a functional crosstalk between these domains [26]. The rationale behind the design of VEEV nsP2pro-2 (436–785) was that there is a strong sequence conservation in the midst of the segment between residue 436 and 457 and it is possible, that this segment is structurally intertwined with the protease domain (Figure S1). Upstream to this segment, the 357–436 region is the C-terminus of the most highly conserved region of the protein (corresponding to a RecA-like nucleotide-binding domain but also annotated as a helicase superfamily type I domain). Both VEEV nsP2 and nsP2pro-2 were terminated at Thr785, which is considered as the end of the density in the current structure. The ORFs of VEEV nsP2 and VEEV nsP2pro-2 coding for 1–785 and 436–785 residues, respectively, were cloned into both pDEST17 and pDEST-His_6_-MBP destination vectors according to Tropea et al. [27].

### 2.2. Expression and Purification of VEEV PR Constructs

**Figure 2:**
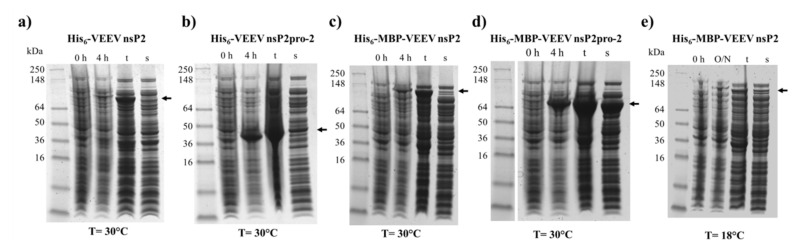
Expression and solubility profiles of VEEV nsP2 and nsP2pro-2. Samples represent the expression profile before (0 h) and after 4 h (4 h) or overnight (O/N) incubation of the non-permeabilized IPTG-induced cells, and the total (t) and soluble (s) protein fractions after cell permeabilization. (**a**) hexahistidine (His_6_)-VEEV nsP2, (**b**) His_6_-VEEV nsP2pro-2, (**c**) His_6_-maltose binding protein (MBP)-VEEV nsP2, and (**d**) His_6_-MBP-VEEV nsP2pro-2 were expressed at 30 °C, while (**e**) His_6_-MBP-VEEV nsP2 was expressed at 18 °C in *E. coli* Rozetta cells. Arrows show the recombinant proteins, the expected molecular weights are listed as follows. His_6_-VEEV nsP2: 91.7 kDa, His_6_-VEEV nsP2pro-2: 43.3 kDa, His_6_-MBP-VEEV nsP2: 131.3 kDa, and His_6_-MBP-VEEV nsP2pro-2: 83.0 kDa.

The His_6_- and His_6_-MBP-fused VEEV nsP2 and nsP2pro-2 recombinant proteins were expressed in *E. coli* Rozetta cells. We have compared the expression and solubility profiles of the different protein forms (Figure 2). For VEEV nsP2pro-2, both the His_6_- and the His_6_-MBP fusion forms were overexpressed by the IPTG-induced bacterial cells after 4 h of incubation at 30 °C, but the His_6_-MBP-fused form showed substantially higher solubility (Figure 2b,d). These findings are in agreement with the previously reported effect of an N-terminal MBP-fusion tag that was found to improve water-solubility of the recombinant proteins and facilitate proper folding [28,29,30]. In the case of VEEV nsP2, the His_6_- and His_6_-MBP-fused constructs were hardly detectable in soluble fractions and showed relatively lower expression levels as compared to VEEV nsP2pro-2 constructs (Figure 2a,c). In order to improve expression and/or solubility of His_6_-MBP-VEEV nsP2, lower incubation temperature (18 °C) and longer incubation time (overnight) were also applied, but these conditions failed to improve the level of expression (Figure 2e).

**Figure 3:**
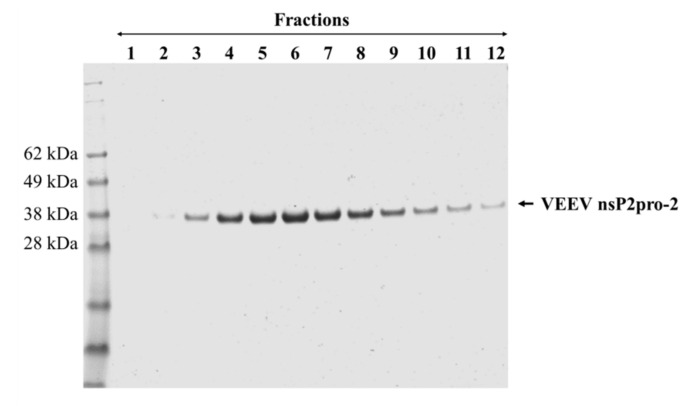
Purification of tag-removed VEEV nsP2pro-2 by gel filtration. The peak fractions (1–12) containing the protease were collected and analyzed by reducing SDS-PAGE. Fractions 2–12 were pooled and concentrated to 2.9 mg/mL final concentration. Arrow shows VEEV nsp2pro-2.

Although, the experimental conditions were not further investigated extensively, we assume that the low solubility of His_6_-VEEV nsP2 was caused by inherent structural properties and folding. The solubility may have been possibly improved by the attachment of MBP, similarly to His_6_-MBP-VEEV nsP2pro-2, however, the conjugation of the VEEV nsP2 with MBP resulted in a fusion protein with a relatively large size (131.3 kDa). This may overwhelm the advantageous effect of MBP on the folding, thus attachment of MBP possibly prevented successful bacterial expression in *E. coli* Rozetta cells at the investigated fermentation conditions. By considering the expression and solubility profiles of the different VEEV PR constructs, we performed downstream experiments only with the His_6_-MBP-fused form of VEEV nsP2pro-2. After affinity purification of the fusion protein, the N-terminal His_6_-MBP dual-tag was removed enzymatically using a His_6_-tagged TEV PR. The untagged VEEV nsp2pro-2 was recovered from the flow-through after a second immobilized metal chelate affinity chromatography step, and then the protease was further purified by gel filtration (Figure 3). The final product was found to have ≥95% purity as determined by SDS-PAGE.

### 2.3. Cloning of Recombinant Fluorescent Substrate-Expressing Vectors

The activity of VEEV nsP2pro-1 has been demonstrated previously in vitro using a synthetic oligopeptide substrate that represented P6-P6′ residues of the wild-type nsP12 cleavage site of SFV (EYHAGA↓GVVETP) [20]. In this study, we applied recombinant fusion protein substrates to measure VEEV nsP2pro-2 activity, the substrates represented either the wild-type or modified SFV nsP12 recognition site.

First, an “empty” expression plasmid (pDEST-His_6_-MBP-mEYFP) was generated that contained the coding sequences of His_6_- and MBP fusion tags, followed by a TEV PR cleavage site (ENLYFQ↓G), a “cloning cassette”, and a C-terminal mEYFP. The generation of the cleavage site-coding dsDNA sequences to be inserted into the empty vectors were performed either directly via the hybridization of complementary, *E. coli* codon-optimized oligonucleotide primers as described earlier by Bozóki et al. [23,24] or by random mutagenesis of a wild-type SFV nsP12 cleavage site. The process for the generation of a His_6_-MBP-fluorescent protein substrate library by random mutagenesis is illustrated in Figure S2.

For characterization of the protease, we designed such substrates which were substituted at P5, P4, P2, P1, or P2′ positions of the SFV nsP12 cleavage site, furthermore, all possible P1′ variants were prepared for the screening of P1′ specificity.

### 2.4. Expression and Purification of the Recombinant Substrates

All His_6_-MBP-mEYFP recombinant substrate variants (Table 1a) were expressed in *E. coli* BL21(DE3) cells and purified based on the protocol described previously [23,24]. For the microplate-based specificity screening, the expression volume was reduced to a final volume of 15 mL.

**Figure 4:**
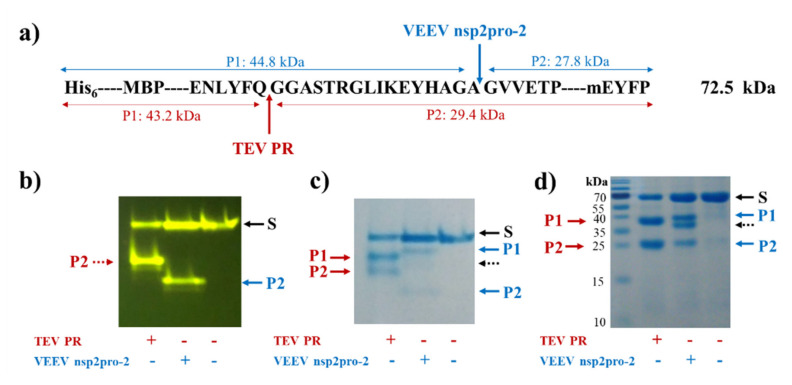
Cleavage of His_6_-MBP-EYHAGA↓GVVETP-monomeric enhanced yellow fluorescent protein (mEYFP) substrate. (**a**) The cleavage site sequence inserted into the His_6_-MBP-mEYFP substrate is shown. Cleavage positions of tobacco etch virus protease (TEV PR) and VEEV nsP2pro-2 are shown by red and blue arrows, respectively. Calculated molecular weights of cleavage fragments (P1 and P2) are also indicated. Cleavage reactions were followed by electrophoretic analysis of mixtures using native (**b**,**c**) or reducing conditions (**d**). The bands were detected in the gels by blue light trans-illumination (**b**) and by Coomassie staining (**c**,**d**). Black dashed arrows show the band of VEEV nsP2pro-2.

The recombinant His_6_-MBP-EYHAGA↓GVVETP-mEYFP protein—containing the wild-type cleavage site sequence—was digested by TEV PR and VEEV nsP2pro-2, as well. Cleavage reactions proved the susceptibility of the protein substrate for proteolysis, furthermore, the yellow fluorescence detected the full-length substrate and C-terminal cleavage products indicated proper folding of mEYFP (Figure 4).

### 2.5. Microplate-Based Specificity Screening of VEEV nsP2pro-2

The substrate specificity of the untagged VEEV nsP2pro-2 was tested using the His_6_-MBP-EYHAGA↓GVVETP-mEYFP recombinant substrate variants, by adapting the previously described Ni-NTA agarose bead-based protease assay to 96-well plates [24].

**Figure 5:**
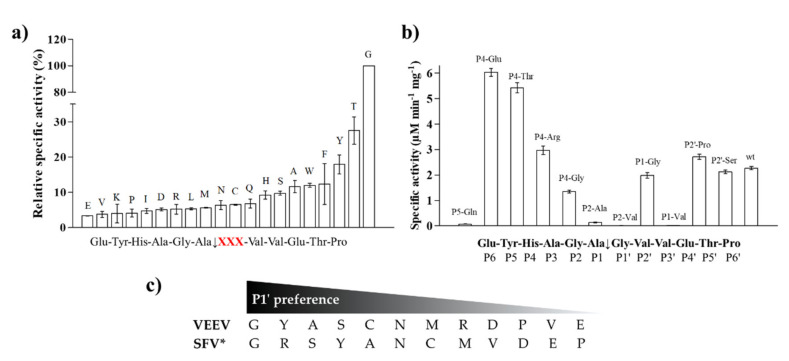
Specific activity of VEEV nsP2pro-2 measured on His_6_-MBP-mEYFP variants. (**a**) Screening of substrates containing P1′ variations of the wild-type Semliki forest virus (SFV) nsP12 (EYHAGA↓GVVETP) recognition site. Relative specific activity values (%) are shown in increasing order, activity determined for the wild-type substrate was considered to be 100%. P1′ residues are labelled in the graph. Cleavage site sequence is shown in the title of x axis where “XXX” denotes modified position (shown in red). (**b**) Values determined for substrate variants comprising substitutions at P5, P4, P2, P1, and P2′ positions. Sequence of cleavage site is also shown. Error bars represent SD, *n* = 2. (**c**) Comparison of P1′ preferences of VEEV and SFV proteases. P1′ preferences determined for VEEV nsP2pro-2 are shown based on figure part (**a)** * Preferred P1′ residues are ordered from best to worst in the case of SFV protease based on Lulla et al. [32]. Substrates representing nsP1/nsP2 or nsP3/nsP4 were used to study specificity of VEEV or SFV protease, respectively.

First, screening of P1′ variants was performed (Figure 5a). The results showed highest preference for a P1′-Gly residue that correspond to the wild-type P1′ residue of EYHAGA↓GVVETP cleavage site, while significantly lower relative activities were determined for all other P1′ variants (Figure 5a).

Some P5, P4, P2, P1, and P2′ mutants were also tested, including P5-Gln, P4-Glu, P4-Thr, P4-Arg, P4-Gly, P2-Ala, P2-Val, P1-Gly, P1-Val, P2′-Pro, and P2′-Ser. Significantly higher activities were obtained for the P4-Glu and P4-Thr variants as compared to that of the wild-type. The specific activities measured on P4-Arg, P1-Gly, P2′-Pro and P2′-Ser variants were comparable to that of the wild-type, but the differences were found to be significant in each case. We observed significantly lower specific activity on the P4-Gly variant compared to the wild-type and only negligible turnover for P5-Gln, P2-Ala, P2-Val, and P1-Val mutant substrates (Figure 5b). The latter results imply that these residues are not preferred in these positions, at least in the context of this cleavage site. The results of one-way ANOVA were in accordance with that of the unpaired *t*-test in all cases. We compared the P1′ specificity of VEEV nsP2pro to that of SFV PR: the amino acid preferences of SFV were determined previously using 12 substrates that represented either the wild-type or 11 P1′ variants of SFV nsP3/nsP4 cleavage site [31]. The P1′ preferences of VEEV and SFV proteases are highly similar: both enzymes tolerate a wide range of P1′ residues and were found to have preference for P1′-Gly (Figure 5c). Both enzymes showed higher cleavage efficiency in the case of mutants containing Tyr, Ser, or Ala in P1′ position, while Asp, Pro, Val, and Glu residues were less preferred.

### 2.6. Kinetic Studies of VEEV nsP2pro-2

**Figure 6:**
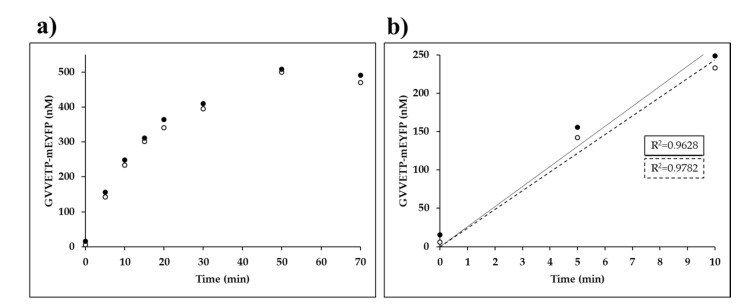
Time-course kinetic measurements of VEEV nsP2pro-2. (**a**) The amount of the GVVETP-mEYFP generated from wild-type His_6_-MBP-EYHAGA↓GVVETP-mEYFP (at a 21.2-µM final concentration) by VEEV nsP2pro-2 (at a 3.0-µM final total concentration) is plotted as a function of time. In the graphs, results of two independent experiments are plotted and the points of datasets are shown by full and open circles. (**b**) The 0–10 min timeframe of figure part (**a**) is enlarged, in this timeframe the enzyme activity was considered to be linear with time. For datasets which are shown by full and open circles the regression lines are indicated by a continuous and a dotted line, respectively.

Time-course kinetic analysis was performed by the Ni-NTA magnet bead-based protease assay in microcentrifuge tubes, using the wild-type His_6_-MBP-EYHAGA↓GVVETP-mEYFP substrate (Figure 6).

We observed a substantial decrease in VEEV nsP2pro-2 enzyme activity over incubation at 30 °C. The applied temperature was previously found to be optimal for VEEV nsP2pro-1 [20], thus we applied the same condition for VEEV nsP2pro-2. We observed a decrease in enzyme activity in the time-course assay, but this is not unusual and similar thermal stability has already been observed previously for SFV nsP2 alphaviral protease [33]. Time-course assay of SFV nsP2 PR activity also showed a decrease in activity at >10 min incubation at 30 °C [33]. The enzyme was incubated in cleavage buffer followed by SDS-PAGE analysis but we observed no appearance of any bands in the gel even after overnight incubation, which implied that the enzyme does not undergo autolysis or degradation during enzyme reactions.

Based on the result of our analysis (Figure 6) we also set the reaction time to 10 min in order to ensure linear correlation between incubation time and product formation for substrate-dependent kinetic measurements and reliable determination of initial velocity at different substrate concentrations. Consequently, kinetic parameters of VEEV nsP2pro-2 could only be determined on those substrates for which cleavage resulted in a reliably detectable fluorescent signal at a concentration below enzyme saturation (at 0.7–70.0 µM), using 10-min-long incubation time. In certain cases the applied conditions resulted in the formation of products only at low concentration (≥0.05 µM), which is close to the lower limit of quantification of the Ni-NTA magnetic-bead based method while mEYFP is used as a fluorescent reporter. Nevertheless, by the application of described reaction conditions k_cat_, K_M_ and k_cat_/K_M_ values for VEEV nsP2pro-2 were successfully determined on the wild-type His_6_-MBP-EYHAGA↓GVVETP-mEYFP substrate.

**Figure 7:**
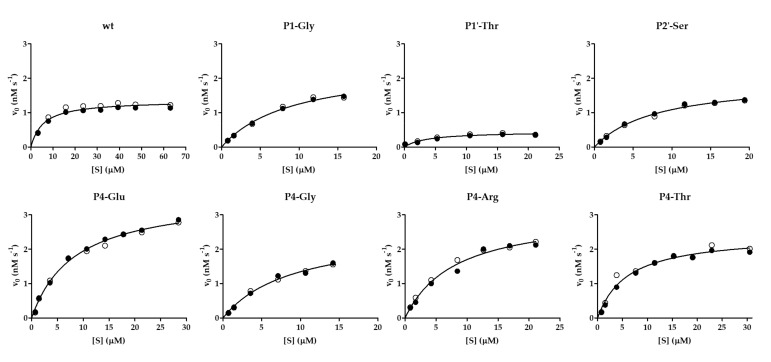
Kinetic curves determined for some cleavage variants. Graphs show results obtained for the wild-type His_6_-MBP-EYHAGA↓GVVETP-mEYFP substrate and its P4-Glu, P4-Thr, P4-Arg, P4-Gly, P1-Gly, P2′-Ser and P1′-Thr variants. The cleavage reactions by VEEV nsP2pro-2 were performed at ≤20% substrate conversion. Results of two independent experiments are plotted and shown by full and open circles. Non-linear regression lines were fitted to average values.

Besides the kinetic characterization of VEEV nsP2pro-2 using the wild-type His_6_-MBP-EYHAGA↓GVVETP-mEYFP substrate, non-linear substrate-dependent kinetic curves were also successfully obtained for P4-Glu, P4-Thr, P4-Arg, P4-Gly, P1-Gly, P1′-Thr, and P2′-Ser variants, that allowed the determination of kinetic parameters (Table 1a). The results resembled those of the microplate-based specificity measurements: VEEVnsp2pro-2 showed the highest catalytic efficiency on P4-Glu variant as compared to the wild-type substrate (~5-fold higher) and this difference was found to be statistically significant. P4-Thr and P4-Arg variants were processed 3.7- and 1.7-fold more efficiently than wild-type SFV nsP12 (Table 1a), respectively, but the difference was statistically significant only in the case of the P4-Thr mutant. The k_cat_/K_M_ values of variants including P4-Gly, P1-Gly, and P2-Ser were calculated to be within the lower error range of the k_cat_/K_M_ value of the wild-type SFV nsP12 recognition site, indicating similarity in their catalytic efficiency. Accordingly, no significant difference was detected by the applied unpaired *t*-test. In contrast, SFV nsP12 P1′-Thr catalytic efficiency was found to be approximately fourfold lower than that of the native sequence, which was considered as a statistically significant difference. The non-linear kinetic curves of the substrate-dependent measurements are demonstrated in Figure 7.

The k_cat_ and K_M_ values were determined to be 0.0003 s^−1^ and 0.0056 mM, respectively, while the k_cat_/K_M_ value was 0.06 ± 0.01 mM^−1^ s^−1^ for VEEV nsP2pro-2, using the substrate that represented the wild-type SFV nsP12 cleavage site (Table 1a). Previously, the catalytic efficiency and the individual kinetic parameters of VEEV nsP2pro-1 have also been determined [5,20], using substrates containing the same recognition site (EYHAGA↓GVVETP) (Table 1b). The catalytic efficiencies of the VEEV nsP2pro-1 and VEEV nsP2pro-2 have the same order of magnitude, while the k_cat_ and K_M_ values obtained for VEEV nsP2pro-2 are remarkably lower as compared to VEEV nsP2pro-1 [5,20]. The observed difference between the K_M_ values may be caused by conformational differences of the substrates, as it was observed earlier in the case of the oligopeptides and His_6_-MBP-FP recombinant proteins which were used for kinetic measurements with TEV PR and human immunodeficiency virus type-1 proteases [23].

It is important to note regarding the interpretation of results that some enzyme kinetic measurements were performed at apparently suboptimal substrate concentrations and the applied substrate concentrations were relatively low compared to the enzyme concentration (see Section 4.9). However, the reactions were monitored at <20% substrate turnover at each substrate concentration in order to ensure measuring initial velocity of product formation and to avoid significant depletion of initial substrate concentration or accumulation of generated products. Furthermore, we assumed that the relative amount of the enzyme was even lower as compared to that of the substrate (K_M_), because the actual active enzyme concentrations were overestimated. The reason for this was that due to the lack of any selective tight-binding inhibitors, the determination of the active site concentration or the folding efficiency of VEEV nsP2pro was not possible, therefore, the activity of the enzyme was regarded as 100%. Considering this assumption, the obtained k_cat_/K_M_ values suggest that the catalytic efficiency of VEEV nsP2pro is similar on different substrate constructs bearing the wild-type SFV nsP12 recognition site. Based on this, the herein presented data imply that in vitro activity of VEEV nsP2pro-1 was not altered substantially by the attachment of the 436-457 region of VEEV nsP2.

### 2.7. Structural Background and Results of In Silico Analyses

#### 2.7.1. Comparison of VEEV and SFV Cleavage Site Sequences

**Figure 8:**
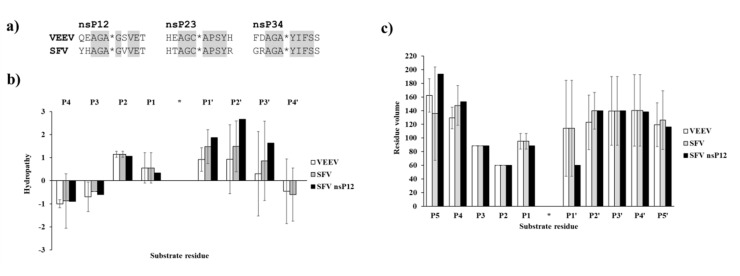
Comparison of VEEV and SFV PR cleavage site sequences. (**a**) nsP12, nsP23, and nsP34 cleavage sites sequences of VEEV and SFV PRs are shown. Identical P3–P4′ residues in the cleavage site sequences are highlighted by grey background. (**b**) Hydropathies were calculated for the cleavage site sequences using the ProtScale module of ExPASy web server, by setting a 3-residue window. For comparison, values for SFV nsP12 cleavage site residues are also shown. (**c**) Residue volumes are shown for P5–P5′ residues of nsP12, nsP23, and nsP34 cleavage sites. For comparison, values for SFV nsP12 cleavage site residues are also shown in both graphs. Error bars represent SD, *n* = 3.

First, the natural cleavage site sequences of VEEV and SFV PRs were compared in order to determine similarities and differences of nsP12, nsP23, and nsP34 cleavage sites. The P3–P4′ residues of VEEV and SFV PR cleavage sites sequences were found to be fully identical, except P2′ position of nsP12 cleavage site. The P3 and P2 residues are identical and are Ala and Gly in each cleavage site sequences, respectively (Figure 8a), while characteristic differences can be observed in P5 and P4 substrate residues in the case of the Old World (e.g., SFV, CHIKV) and New World alphaviruses (e.g., VEEV) [5].

The comparison of VEEV and SFV PR cleavage sites showed that hydropathies of natural nsP12, nsP23, and nsP34 cleavage sites are similar and the investigated sequences contain mainly hydrophobic residues in P2–P2′ positions, while the outer residues (e.g., P4, P4′) are hydrophilic (Figure 8b). Due to the high similarities of the sequences, the residue volumes also resemble each other in VEEV and SFV cleavage sites (Figure 8c). Ala and Gly residues are highly conserved in P3 and P2 positions, respectively, and Ala/Cys can be found in P1 position, thus, average volumes of P3–P1 residues are relatively smaller as compared to other positions.

#### 2.7.2. In Silico Mutation Analysis

A complex of VEEV PR (encompassing 469–767 residues of VEEV nsP2) with EYHAGA↓GVVETP substrate was prepared based on a modeled structure of the enzyme complexed with a LQEAGA↓GSVETP substrate (representing VEEV nsP12 cleavage site sequence). The complex structure was refined by molecular dynamics simulations, the results of the equilibrium simulation are visualized in Figure S3.

The refined complex structure contained a peptide representing the wild-type nsP12 cleavage site sequence of SFV. To investigate the effects of substrate modifications on the enzyme–substrate interactions, the mutations were introduced in silico by using DynaMut web server and the folding free energy changes upon the point mutations (ΔΔG, kcal/mol) were calculated. The predicted values are shown in Table 1a.

The results regarding the studied P5, P4, P2, P1, P1′, and P2′ variants are discussed as follows, including investigation of enzyme–substrate interactions, the results of in silico calculations and their correlation with in vitro measurements, as well.

#### 2.7.3. P5 Specificity

**Figure 9:**
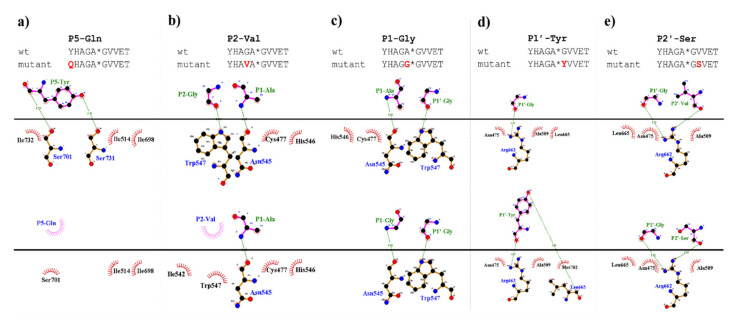
Comparison of interaction maps for wild-type and some mutant substrates. The mutant complexes were prepared using DynaMut web server. Upper parts of the figures show interactions for the wild-type substrates, while bottom parts for the P5-Gln (**a**), P2-Val (**b**), P1-Gly (**c**), P1′-Tyr (**d**), and P2′-Ser (**e**) mutants. Figures were prepared by plotting protein–protein interactions using DIMPLOT module of LigPlot+ v2.1.

In vitro measurements showed only marginal substrate turnover for the P5-Gln mutant as compared to the wild-type substrate (Figure 5). The substitution of P5-Tyr to Gln was predicted to be destabilizing (ΔΔG = −0.222 kcal/mol), therefore, weakening enzyme–substrate interactions. The more preferable binding of a Tyr residue at S5 site can be explained by the formation of a H-bond between hydroxyl group of P5-Tyr and Ser-731, and between a main-chain atom of P5-Tyr with a Ser-701 side chain, interactions that were not predicted for the P5-Gln mutant (Figure 9a).

#### 2.7.4. P4 Specificity

The S4 site was previously found to consist of polar and nonpolar residues and mainly polar enzyme–substrate interactions were dominant [31]. Structural analyses revealed that Lys-706 residue makes a salt bridge with a P4-Glu side chain, and this site was supposed to be responsible for the recognition of acidic P4 side chains [5,31].

As it was expected, we measured higher in vitro proteolytic activity on P4-Glu variant as compared to the wild-type substrate. This Glu in P4 position corresponds to a wild-type acidic residue of VEEV nsP12 and nsP23 cleavage sites (Figure 8a). The P4-Thr and P4-Arg mutants were also cleaved more efficiently as compared to the wild-type. Out of the tested P4 variants, the lowest catalytic constant was measured for P4-Gly mutant, however, the obtained value was comparable with that of the wild-type substrate (Table 1a). The observations can be explained by the lack of side-chain-mediated polar interactions at S4 site in the case of the P4-Gly mutant.

The stability analysis upon point mutations resulted in stabilizing changes in the P4-Glu mutant only. In agreement with this, among all the studied SFV nsP12 variants, the highest activity was determined for this substrate, the k_cat_/K_M_ was approximately fivefold higher than for the wild-type substrate (Table 1a).

**Figure 10:**
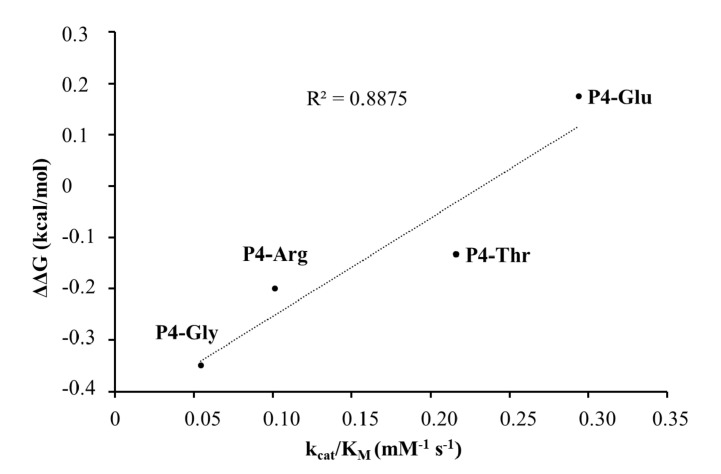
Correlation between the calculated ΔΔG values and the in vitro k_cat_/K_M_ values.

In the case of the studied P4 variants, the predicted changes of free energy showed good correlation with the in vitro obtained k_cat_/K_M_ values (Figure 10).

#### 2.7.5. P2 Specificity

The activities determined for the P2-modified substrates were highly similar to each other and were considerably lower as compared to the wild-type (Figure 5). The predicted energy changes showed no correlation with the in vitro observed effects of the mutations, as we predicted increased and decreased free energy for P2-Val and P2 Ala mutants, respectively (Table 1a). In P2 position, VEEV cleavage sites contain a Gly residue which is highly conserved across alphavirus species (Figure 8a). Based on the results we propose that larger Ala or Val residues are less preferred as this site. As it was predicted, the hydrogen bond formed by a main-chain atom of P2-Gly is not preserved either in the P2-Val (Figure 9b) or in the P2-Ala mutant, which may contribute to lower preference for binding Val or Ala at S2 site.

#### 2.7.6. P1 Specificity

Mainly Ala, Cys, and Arg residues can be found in P1 positions of nsP12, nsP23, and nsP34 alphaviral cleavage sites (Figure 8a), while Gly is present only in nsP34 sites of a few viruses, e.g., in SINV and CHIKV [21].

We studied the effects of the substitution of P1-Ala residue to Gly and Val, and we found that both residues are less preferred in P1 position as compared to the wild-type, and we observed only marginal processing of P1-Val mutant (Figure 5). Covariances of residues implied relatively higher tolerance of this site towards amino acid substitutions [31], and in agreement with this, we found that the obtained k_cat_/K_M_ values for the wild-type and the P1-Gly mutant substrates are comparable, but higher K_M_ was determined for the latter one (Table 1a).

Based on the structures, hydrophobic S1-P1 interactions may be more favorable in the case of P1-Ala residue (Figure 9c), which is in agreement with the slightly higher catalytic constant determined for the wild-type as compared to the P1-Gly mutant.

Interestingly, the Val residue—with a larger volume and higher hydrophobicity as compared to Ala—was not preferred at P1 site (Figure 5). This can be explained in part by the observations of Russo et al. [31], who found that the branched side chains have significantly lower flexibility that makes their binding to the S1 site less favorable. Although, the predicted changes implied more preferable interactions for P1-Val mutant than for the P1-Gly mutant (Table 1a), processing of P1-Val mutant was less efficient in vitro. This indicates that the applied in silico prediction method is not sensitive enough to estimate structural changes and side-chain flexibility accurately for all mutants. Extended molecular dynamics calculations may be used to predict changes of mutations more efficiently, but the application of such calculations for all mutants was out of the scope of this study.

#### 2.7.7. P1′ Specificity

The activities obtained in vitro for the P1′-modified substrate series were correlated with the hydrophobicities and volumes of the individual P1′ residues and with the overall hydrophobicity profiles of cleavage sites.

**Figure 11:**
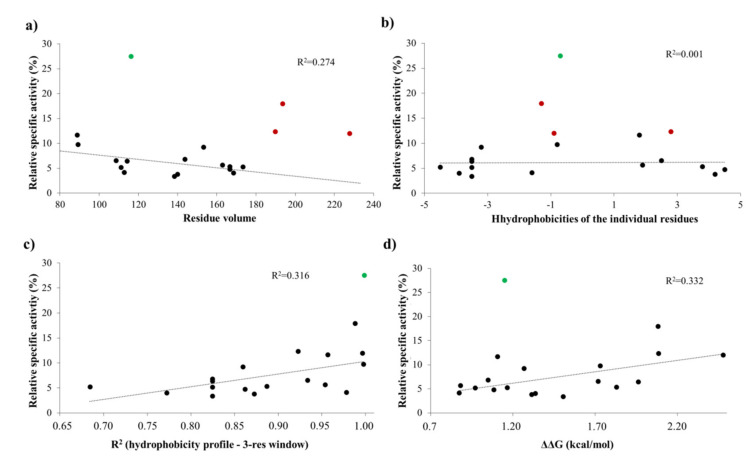
Dependence of activity on different cleavage site features. The relative activities were plotted as a function of the volume (**a**) and hydrophobicity of each individual P1′ residue (**b**), the overall hydrophobicity of the cleavage site (**c**), and the ΔΔG value calculated for all P1′ mutant (**d**). Values obtained for the wild-type substrate (P1′-Gly) were not plotted (relative specific activity: 100%). The values that were used for the correlation are shown by black dots, and those values which were excluded from the linear regression are colored as follows: green, P1′-Thr; red, P1′-Tyr, P1′-Trp, and P1′-Phe.

Our results imply that there is no direct correlation between the P1′ specificity and the hydrophobicity (Figure 11a) or volume (Figure 11b) of P1′ individual residues, rather the overall hydrophobicity of the entire cleavage site may be a determinant for the specificity (Figure 11c). Our results are in agreement with those of Russo et al. [31], who found that the enzyme forms contacts mainly with the main-chain atoms of the substrate at the S1’ site, but the interactions seem to be not independent from the side chain. Besides Gly, the most preferred P1′ residues have mainly polar (Thr, Ser) or aromatic (Tyr, Trp, Phe) side chains. In agreement with our comparison (Figure 8a), all nsP12, nsP23, and nsP34 natural cleavage site sequences of alphaviruses were found previously to contain Gly, Ala, or Tyr in P1′ position [32]. Out of these three residues, we observed highest activity for P1′-Gly (wild-type) and P1′-Tyr variants, while cleavage of P1′-Ala mutant was less efficient (Figure 5a). In this position, a P1′-Tyr residue can form a H-bond interaction with a main-chain atom of Leu-665 residue (Figure 9d). The aromatic rings enable favorable hydrophobic interactions in the case of P1′-Trp and P1′-Phe mutants, as well, therefore, enable preferable binding of the Phe, Tyr, and Trp residues at this site. In agreement with this, most remarkable changes of ΔΔG (>2 kcal/mol) were predicted for these mutants (Table 1a).

The changes of folding free energy (ΔΔG) values predicted for the P1′-mutant series also showed no strong correlation with the results of in vitro specificity screening (Figure 11d).

We found that the substrates containing basic or acidic P1′ residue (but not His) showed lowest (<5%) substrate conversion, in the context of SFV nsP12 cleavage site (Figure 5).

Besides the wild-type substrate, highest activity was obtained for the P1′-Thr mutant. In accordance with the microplate-based screening results, a threefold lower catalytic constant was obtained for P1′-Thr mutant compared to the wild-type, although predictions showed no such changes of energy for this mutant (Figure 11d).

#### 2.7.8. P2′ Specificity

The nsP23 and nsP34 alphaviral (VEEV, EEEV, WEEV, SFV, CHIKV, and SINV) cleavage sites contain hydrophobic/non-polar residues (Pro, Leu, Ile, Val) in P2′ position, while the polar Ser can be found in nsP12 cleavage sites of VEEV, EEEV, and WEEV (QEAGA↓GSVET).

In this study, we have substituted the wild-type Val residue in P2′ position of SFV nsP12 cleavage site (EYHAGA↓GVVET) to Ser (Figure 9e) and to Pro, however the kinetic parameters were only determined in the case of the Ser variant.

**Figure 12:**
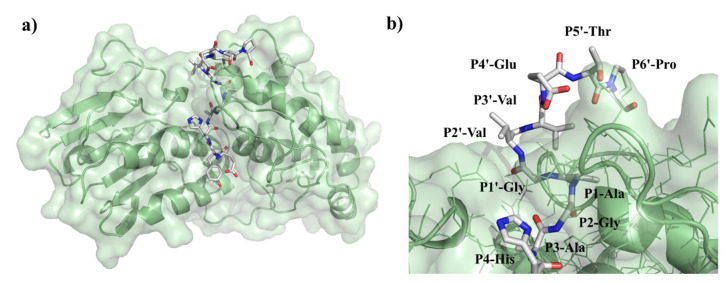
Structure of VEEV PR complexed with EYHAGA↓GVVETP substrate. (**a**) The overall structure of the modeled complex is represented. (**b**) Enlarged view of the active site with the bound substrate. Figure shows that P2′ residue of the substrate is exposed to the solvent.

Previously performed specificity studies revealed that mainly S4-S1′ sites contribute significantly to substrate recognition [31,32]. These sites are well-characterized, but the information about P2′ site is limited. The S2’ site is a less well-defined pocket compared to S4-S1′ sites: the P2′–P6′ residues of the bound substrate are exposed to the solvent (Figure 12), but previously modeled enzyme–substrate complexes suggested favorable backbone interactions for P1′–P6′ sites [5].

Hydrophobicity of the SFV nsP12 wild-type P2′ residue (Val) is higher than that of the Ser mutant, the volumes of these residues are almost identical. Despite the different side-chain characteristics, the k_cat_/K_M_ determined for the wild-type and P2′-Ser mutant substrates were almost identical (Table 1a). Thus, in agreement with the previous findings our results also imply that S2’–P2′ interactions are mainly backbone-mediated, and the side chain of a P2′ residue is not recognized specifically by side-chain-mediated contacts at this site.

Stability analysis implied slightly lower interaction energy between the enzyme and the substrate upon P2′-Ser mutation, while increased energy was predicted for P2′-Pro mutation (Table 1a). In contrast, activities determined in vitro by the two P2′ site-mutants were similar and comparable with that of the wild-type (Figure 5). In the case of the P2′-Ser variant the k_cat_/K_M_ values were practically identical to that of the wild-type and both k_cat_ and K_M_ was ~1.5-fold higher for the P2′-Ser mutant (Table 1a). The in vitro results show correlation with the fact that the P2′ side chain is exposed to the solvent, and the Ser side chain in this position forms no H-bonds (Figure 9e).

## 3. Discussion

This study was made with the aim of characterization of nsP2 cysteine protease of VEEV. First, we compared the expression profiles and solubility of two N-terminally elongated constructs. We studied a nearly full-length VEEV nsP2 (1–785) and a VEEV nsP2pro-2 (436–785) construct (Figure 1). The VEEV nsP2pro-2 construct contains the C-terminal protease domain of nsP2 extended with the C-terminal segment of the N-terminal domain (436–457). The recombinant proteins were fused either to His_6_- or His_6_-MBP tags and expressed in *E. coli* Rozetta cells. We found a low level of expression and marginal solubility for both the His_6_- or His_6_-MBP-fused VEEV nsP2 in each tested condition. In the case of the His_6_-VEEV nsP2, this may be explained in part by the inherent structural and folding properties of the protein. Based on the findings of Saisawang et al. [34], the insufficient solubility may be a result of the aggregation of the construct with the bacterial host cell proteins. As they described, the attachment of MBP to the full-length CHIKV nsP2 solved the problem related to inclusion body formation, but the tagged proteins were still aggregated with contaminating *E. coli* proteins. In contrast, the expression and solubility level of His_6_-MBP-VEEV nsP2pro-2 construct was sufficient (Figure 2). Therefore, we used this construct to prepare VEEV nsP2pro-2 for enzyme specificity studies. The untagged VEEV nsP2pro-2 was produced in high purity using a four-step purification protocol including an enzymatic removal of the dual fusion tag by TEV protease (Figure 3).

The enzyme activity measurements were performed by using a recombinant fluorescent protein substrate-based protease assay, which has been previously applied successfully to study TEV and HIV-1 proteases [23], the retroviral-like protease of human paternally expressed gene 10 (PEG10) protein [35] and yeast retrotransposon Ty1 [36]. The previously developed method was slightly modified and some improvements were introduced as follows. We reduced the volume of substrate expression from 50 to 15 mL, the small-scale expression was carried out in centrifuge tubes that enabled working with multiple mutants simultaneously. The protease assay was adapted to a 96-well plate-based format, reduced volumes of working samples increased cost-efficiency and throughput of the assay as compared to the microcentrifuge tube-based assay. Random mutagenesis was also used for the generation of the mutants, which has not been applied previously in the case of this assay system.

We have adapted the previously designed substrate system to investigate VEEV nsP2 protease, and generated His_6_-MBP-mEYFP substrates that contained the wild-type or modified nsP12 cleavage site of SFV (EYHAGA↓GVVETP). The complete series of P1′ variants and other substrate mutants may be applicable to study additional alphavirus proteases, e.g., specificities of SFV and CHIKV PRs. Substrates containing SFV nsP12 recognition sequence were chosen for VEEV PR enzymatic assays because substrates representing the same cleavage site have already been applied successfully for the investigation of VEEV nsP2 protease [5,20], which is supported by our earlier specificity results which suggested that the peptide representing SFV nsP12 was a substantially better substrate of VEEV protease than its cognate sequences [20]. The designed recombinant proteins were proved to be folded properly and are susceptible for cleavage by TEV and VEEV nsP2 proteases (Figure 4). The generated substrate library can be potentially used to study other alphaviral proteases because, e.g., proteases of SFV, SINV are also known to cleave SFV nsP12 cleavage site [20].

The kinetic parameters obtained for VEEV nsP2pro-2 on the protein substrate containing nsP1/nsP2 cleavage site of SFV, were compared to the kinetic data determined previously for VEEV nsP2pro-1 using oligopeptide substrates [20] or CFP/YFP-based recombinant FRET substrates [5] coding the same cleavage site sequence. The differences between the K_M_ values may be explained by the conformational differences of the different substrate, in agreement with our previous kinetic studies on TEV PR and HIV-1 PRs [23,24]. The k_cat_ and k_cat_/K_M_ values are not fully comparable with the literature data [5,20], because the active enzyme concentration cannot be determined due to the lack of any selective inhibitor. Still, if enzyme activity is considered as 100% in each case, the k_cat_/K_M_ values are at the same order of magnitude, indicating that the catalytic efficiency of VEEV nsP2pro-2 is similar to that of VEEV nsP2pro-1. This implies that N-terminal extension of VEEV nsP2pro-1 (with 436–457 region of nsP2) has no substantial effect on the activity of the VEEV protease domain in vitro.

To study enzyme specificity of VEEV nsP2pro-2 in the context of nsP12 cleavage site of SFV, we designed His_6_-MBP-mEYFP substrates modified in P5, P4, P2, P1, P1′, and P2′ positions. The screening studies were performed by the 96-well plate-adapted format of the previously designed microtube-based assay [23,24,25]. As the amount of substrate needed for the plate-based format was much smaller than that of the microvial-based setup, we optimized the expression of the substrates to a smaller scale (15 mL), accordingly. Following the microplate-based specificity studies, kinetic parameters were determined for some selected variants. The activities determined by the 96-well plate-based assay were in agreement with the values obtained by the microvial-based measurements. This suggests the utility of the plate-based setup in ranking the hits according to their catalytic efficiency if the applied substrate concentration is in the dynamic range of the enzyme, although the results of the applied statistical tests on the specific activity and k_cat_/K_M_ data are contradictory in the case of P4-Arg, P4-Gly, P1-Gly, and P2-Ser.

To support the interpretation of the in vitro enzymatic measurements, modeled structure of the VEEV nsP2pro (469–767) complexed with the studied oligopeptide substrate was prepared, and the effects of mutations on enzyme–substrate interactions were investigated in silico. Screening of P1′ specificity showed the highest preference of VEEV nsP2pro for a P1′-Gly residue, while significantly lower preference was determined for all P1′ variants in the context of EYHAGA↓GVVETP cleavage site (Figure 5). Structural analysis showed no correlation of the obtained activities with hydrophobicities and volumes of the individual P1′ residues, or with the overall hydrophobicity profiles of cleavage sites, furthermore, predicted stability changes upon the mutations also showed no strong correlation with the in vitro data (Figure 11). Nevertheless, our results are in agreement with the literature data, as previous studies also found that enzyme–substrate interactions at this site are mediated mainly by the main-chain atoms of the substrate [31]. The most preferred variants for which the obtained activity was ≥10% contained Phe, Tyr, Trp, Ser, Thr, or Ala residues in P1′ position (Figure 5). Some P5, P4, P2, P1, and P2′ mutants were also tested in vitro and in silico (Table 1a) (Figure 5). We found significantly lower preference for P5-Gln mutant as compared to the wild-type substrate; we assume that the side chain of the wild-type P5-Tyr forms H-bonds with S5 residues, while the same interactions are not enabled in the case of the P5-Gln variant.

The highest catalytic efficiency was obtained for VEEV nsP2pro-2 by using P4-Glu mutant substrate, in which Glu represents the wild-type P4 residue of VEEV nsP1/nsP2 and nsP2/nsP3 cleavage sites, and its side chain is supposed to connect to Lys-706 of the S4 subsite via a salt bridge [5,31]. For the other tested P4 variants (Thr, Arg, and Gly) the specific activity was significantly lower as compared to P4-Glu, most probably due to the lack of side-chain-mediated polar interactions at S4 site. Nevertheless, P4-Thr variant was processed with significantly higher efficiency in contrast to the wild type, while for P4-Arg and P4-Gly this difference was not significant. The in vitro determined k_cat_/K_M_ values of the different P4 variants were in agreement with the previously reported dominance of polar interactions at this site [31], and showed good correlation with the in silico predicted changes of energy (Figure 10). SFV PR was found previously to be highly specific for P4-Arg which cannot be replaced by other residues in this position [32]. While a P4-Thr residue prevented substrate processing by SFV PR [32], we found that VEEV nsP2pro can cleave a substrate containing Thr in P4 position, thus, our results reveal that S4 site of VEEV nsP2pro has a wider tolerance as compared to that of SFV protease.

In the P2 position, wild-type VEEV cleavage sites contain a Gly residue, which is highly conserved across alphavirus species [21] and is recognized by the “glycine specificity motif” of VEEV nsP2pro, similarly to the mechanism described for other cysteine proteases [19]. The in vitro-specific activity rates for both P2-Val and P2-Ala variants were marginal, most probably owing to (i) the relatively larger sizes of Ala and Val residues and (ii) the lack of a H-bond formed by the backbone atom of Gly.

The specific activity of the P1-Gly variant was half that of the P1-Ala, that may be explained by the preference of hydrophobic S1-P1 interaction. Val has a larger volume and higher hydrophobicity compared to Ala, and accordingly, in silico analysis predicted more favorable interactions for this variant than for Gly; however, the in vitro-measured specific activity on this variant was marginal. This can be explained by the findings of Russo et al. [31], that branched side chains have lower flexibility that is likely to cause less favorable binding to the S1 site. Additionally, they observed that covariances of residues suggest relatively high tolerance for substitutions at P1 site. In accordance with this, the in vitro-determined k_cat_/K_M_ values did not differ significantly between the wild-type and the P1-Gly variants of SFV nsP12 cleavage site.

Both Lulla et al. [32] and Russo et al. [31] described that the substrate is mainly recognized by the S4-S1’ subsites of VEEV nsP2 protease, therefore, previous specificity studies focused mainly on the P4-P1′ preferences. Despite the importance of these sites, the complete screening of P1′ specificity has not been performed experimentally to date. Additionally, the data on the amino acid preferences of S2’ site are also limited. Based on the modeled complex, S2’ site is not a less well-defined pocket as P2′–P6′ substrate residues are more exposed to the solvent. The specific activities determined for P2′-Ser and P2′-Pro variants in vitro were comparable with the values obtained for the wild-type substrates containing P2′-Val residue, and the *k*_cat_/K_M_ constants determined for the wild-type and P2′-Ser substrates were practically identical, despite differences of the hydrophobicities of these residues. This is in agreement with the results of Hu et al. [5] who proposed favorable backbone-mediated interactions for the P1′–P6′ sites.

In summary, our results are in agreement with the literature data and complement knowledge about in vitro specificity of VEEV nsP2pro. While former studies used different approaches to study specificity (e.g., substrate-binding sites of the protease were targeted by mutagenesis [5] or information about specificity was obtained based on different cleavage efficiencies of natural cleavage sites [20]), here we prepared series of modified substrates for position screening. Furthermore, structural backgrounds of enzyme–substrate interactions were studied previously by others [31], and the present study provided experimental evidence for the former findings. Considering the importance of alphaviruses from both economic and military aspects and the lack of any effective therapy against their infections, obviously there is a great demand for the further investigation of these viruses. Thus, our results may provide valuable information about amino acid preferences that may support future studies on inhibitor development against alphaviruses or other important Group IV viruses.

## 4. Materials and Methods

All materials were purchased from Sigma-Aldrich (St. Louis, MO, USA), unless otherwise indicated.

### 4.1. Cloning of VEEV nsP2 and VEEV nsP2pro-2 Expression Vectors

Plasmid encoding the cDNA of non-structural proteins of VEEV was a gift from Dr. Christine L. Pugh (United States Army Research Institute of Infectious Diseases). The ORFs of VEEV nsP2 coding for residues 1–785, and VEEV nsP2pro-2 encoding residues 436–785, have been cloned into both pDEST17 and pDEST-His_6_-MBP destination vectors by Gateway Cloning Technology (Thermo Fisher Scientific, Waltham, MA, USA) according to Tropea et al. [27]. The linear DNA sequences were amplified as follows: (i) The ORF of VEEV nsP2 was amplified using PE2685 and PE2686 primers for PCR according to the following reaction setups: 10 ng template, 5 nmol PE2685, 5 nmol PE2686, 10 µL Phusion Flash High Fidelity PCR Master Mix (New England Biolabs, Ipswich, MA, USA) and nuclease-free water up to 20 µL using the following PCR protocol: cycle1 (1×): 60 s at 98 °C; cycle2 (30×): 15 s at 98 °C, 75 s at 72 °C, and 60 s at 72 °C; cycle3 (1×): store at 4 °C. (ii) The ORF of VEEV nsp2pro-2 was amplified using PE2687 and PE2686 primers for PCR, according to the following reaction setups: 10 ng template, 5 nmol PE2685, 5 nmol PE2686, 10 µL Phusion Flash High Fidelity PCR Master Mix and nuclease-free water up to 20 µL using the following PCR protocol: cycle1 (1×): 60 s at 98 °C; cycle2 (30×): 10 s at 98 °C, 30 s at 72 °C, and 60 s at 72 °C; cycle3 (1×): store at 4 °C. The PCR products were purified by MinElute (Qiagen, Hilden, Germany) and 60–80 ng of the resulting PCR amplicons were subsequently used as the templates for another PCR with 5 nmol PE277, 5 nmol PE2686, 10 µL Phusion Flash High Fidelity PCR Master Mix and nuclease-free water up to 20 µL using the following PCR protocol (i) for VEEV nsP2: cycle1 (1×): 60 s at 98 °C; cycle2 (30×): 15 s at 98 °C, 75 s at 72 °C, and 60 s at 72 °C; cycle3 (1×): store at 4 °C; (ii) for VEEV nsp2pro-2: cycle1 (1×): 60 s at 98 °C; cycle2 (30×): 10 s at 98 °C, 30 s at 72 °C, and 60 s at 72 °C; cycle3 (1×): store at 4 °C. The linear DNA sequences were transferred via pDON221 donor vector (Invitrogen, Thermo Fisher Scientific, Waltham, MA, USA) into pDEST-His6-MBP (Invitrogen, Thermo Fisher Scientific, Waltham, MA, USA) and pDEST17 (Invitrogen, Thermo Fisher Scientific, Waltham, MA, USA) as described by Tropea et al. [27]. The sequence the of entry clones was verified by capillary sequencing. The oligonucleotide primers applied during PCRs and sequencing reactions are listed in Table S1. Expression vectors pBB2546 (pDEST-His_6_-VEEVnsP2), pBB2547 (pDEST-His_6_-VEEVnsP2pro-2), pBB2549 (pDEST-His_6_-MBP-VEEVnsP2), and pBB2550 (pDEST-His_6_-MBP-VEEVnsP2pro-2) were purified by Qiagen Miniprep Kit (Qiagen, Hilden, Germany).

### 4.2. Sequence Alignment

The primary sequences of VEEV nsP2 (NCBI Ref. Seq.: NP_740697.1), SINV (NCBI Ref. Seq: NP_740671.1), CHIKV (GenBank: ADZ47896.1), Sagiyama virus (GenBank: BAA92845.1) and SFV (NCBI Reference Sequence: NP_740666.1) were aligned using multiple sequence alignment tool Clustal Omega (EMBL-EBI, Hinxton, UK).

### 4.3. Generation of the Substrate Expression Plasmids

The generation of the pDEST-His_6_-MBP-mEYFP empty plasmid and the insertion of the cleavage sites of interest to its cloning cassette were performed as described by Bozóki et al. [23,24] using pmEYFP-N1 vector as a template. Plasmid pmEYFP-N1 was generated previously in our laboratory by the modification of EYFP-N1, which was a kind gift of Prof. Thomas Jovin (Göttingen). The modification included the elimination of the EYFP dimerization surface by introducing A208K mutation [37].

The inserted linear short dsDNA sequences coding for wild-type, P5, P4, P1, P2, and P1′ variants of SFV nsP12 cleavage site were generated either by annealing of chemically synthetized complement oligonucleotides primers (Table S2) as described by Bozóki et al. [23,24] or by random mutagenesis on P1′ site as follows. pT7-Blue-3 plasmid (Merck-Millipore, Burlington, MA, USA) was linearized by BamHI and NheI restriction endonucleases (New England Biolabs, Ipswich, MA, USA). Oligonucleotide primers coding for wild-type SFV nsP12 (EYHAGA↓GVVETP) cleavage site sequence that were flanked by BamH I and NheI sticky ends, were inserted into pT7-Blue-3 plasmid. For ligation, 40 ng linearized pT7- Blue-3 plasmid was incubated with 800 ng SFV nsP12 BamHI forward primer (5′-GATCCTTAATTAAAGAGTACCATGCTGGTGCTGGTGTGGTGGAGACACCGG-3′) and 800 ng SFV nsP12 BamHI reverse primer (5′-CTAGCCGGTGTCTCCACCACACCAGCACCAGCATGGTACTCTTTAATTAAG-3′) for 2 min at 65 °C, then for 2 min at 4 °C. Hereafter, T4 DNA Ligase Reaction Buffer (10×) and T4 DNA ligase (New England Biolabs, Ipswich, MA, USA) were added, followed by incubation overnight at 16 °C. An amount of 100 µL of *E. coli* DH5α-competent cells (New England Biolabs, Ipswich, MA, USA) was transformed by 5 µL of the ligation reaction mixture by heat shock (at 42 °C). The transformants were spread on Luria-Bertani (LB) agar plates containing 100 µg/mL ampicillin and were grown at 37 °C overnight. The transformed cells were then cultured in LB medium containing 100 µg/mL ampicillin followed by preparation of pT7-Blue-3-SFV nsP12 plasmid by Qiaprep Spin Mini Prep Kit (Qiagen, Hilden, Germany). Random mutagenesis of the triplets coding for the P1′ residue of SFV nsP12 was performed by QuickChange Lightning Multi-Site Directed Mutagenesis Kit (Agilent Technologies, Santa Clara, CA, USA) according to the manufacturer’s protocol using SFV nsP12 P1′ DEG oligonucleotide primer (5′-GAGTACCATGCTGGTGCTNNNGTGGTGGAGACACCGGCTAGC-3′). P1′-mutagenized pT7-Blue-3 SFV nsP12 plasmids were recovered from the cultures of grown colonies using Qiagen Plasmid Midi Kit (Qiagen, Hilden, Germany) and were linearized using NheI and PacI (New England Biolabs, Ipswich, MA, USA). The short dsDNA sequences coding for SFV nsP12 P1′ variants were separated on polyacrylamide gels containing 15% urea and were purified by Qiaex II Gel Extraction Kit (Qiagen, Hilden, Germany). The mutagenized dsDNA fragment (100 ng) was mixed with 200 ng linearized pDEST-His_6_-MBP-mEYFP plasmid during the ligation reaction described above.

### 4.4. Expression of VEEV nsP2pro-2 and VEEV nsP2

For expression, freezer stocks of *E. coli* Rozetta cells were used that were transformed previously by pBB2546, pBB2547, pBB2549, or pBB2550 plasmids. An amount of 100 µL of freezer stock was added to 100 mL LB medium and incubated 16 h at 37 °C. Hereafter 30 mL of the starter culture was measured to 1000 mL LB medium containing 125 µg/mL ampicillin, 30 µg/mL chloramphenicol and 0.2% glucose. Cells were grown up to an OD_600_ = 0.5–0.6 at 37 °C, and expression was induced by 1 mM isopropyl β-d-1-thiogalactopyranoside IPTG (final concentration) and incubated for 4 h at 30 °C. Cells were harvested at 2000 *g* for 10 min using a tabletop centrifuge. After the removal of the medium, the cell pellets were stored at −80 °C. Other conditions were also tested to improve the expression and solubility of His_6_-MBP-VEEV nsP2, using pBB2549 construct. In this setup, when the 1000-mL culture reached OD_600_ = 0.5, the temperature was shifted to 18 °C, the culture was further incubated for 20 min and after induction and culture was incubated overnight at 18 °C.

To examine protein expression profile of the different constructs, cells were sampled right before induction (0 h), and prior to harvest (4 h or overnight). One ml from each sample was spun at 10,000 *g* for 5 min on a tabletop centrifuge and pellets were stored at −80 °C. The next day after thawing, pellets were re-suspended in distilled water.

For solubility profile analysis, 10 mL suspension right before cell harvest was centrifuged at 5000 *g* for 10 min in a tabletop centrifuge and the pellets were stored at −80 °C. The next day after thawing, cells were lysed with B-PER buffer (1.5 mL B-PER (Pierce, Thermo Fisher Scientific, Waltham, MA, USA), 3 µL lysozyme (2 mg/mL) and 3 µL DNase (New England Biolabs, Ipswich, MA, USA) for 15 min at room temperature. Samples taken from the crude cell lysate represented the total protein fraction, while cleared lysate represented the soluble protein fraction.

Samples were analyzed on 14% SDS-PAGE at reducing conditions.

### 4.5. Purification of VEEV nsP2pro-2

Bacterial cell pellet expressing His_6_-MBP-VEEV nsP2pro-2 construct were suspended in 50 mL of ice-cold buffer A (50 mM sodium phosphate, 150 mM NaCl, 25 mM imidazole, pH 7.5). The cells were disrupted by APV Gaulin Model G homogenizer (Invensys, Albertslund, Denmark) at 10,000 psi and centrifuged at 30,000 *g* for 30 min at 4 °C. The supernatant was filtered through a 0.22-µm polyethersulfone membrane and was applied to a 15-mL (3 × 5 mL) HisTrap FF crude affinity column (GE Healthcare, Piscataway, NJ, USA) equilibrated in buffer A. The elution was performed with a linear gradient from 5–50% buffer B (50 mM sodium phosphate, 150 mM NaCl, 500 mM imidazole, pH 7.5) and eluate fractions containing the His_6_-MBP-VEEV nsP2pro-2 were analyzed by reducing SDS-PAGE. Fractions with the highest concentration were pooled and concentrated with Amicon YM30 membrane (Millipore, Billerica, MA, USA), then diluted 6-fold with 50 mM sodium phosphate buffer (containing 150 mM NaCl, pH 7.5) to reduce the imidazole concentration to 25 mM. The fusion protein was then processed with a 5 mg/mL stock solution of the His-tagged TEV PR (70:1 *v*/*v*) overnight at 4 °C. Hereafter, the cleavage products were applied to a 20-mL (4 × 5 mL) HisTrap FF crude affinity column equilibrated in buffer A. Both flow-through, containing the VEEV nsP2pro protease, and eluate fractions were collected. Flow-through fractions were analyzed on SDS-PAGE at reducing conditions and those that contained a high amount of VEEV nsP2pro-2 were pooled. The sample was concentrated to 10 mg/mL using an Amicon YM30 membrane (Millipore, Burlington, MA, USA) and applied to a HiPrep 26/60 Sephacryl S200 gel filtration column (GE Healthcare, Chicago, IL, USA) equilibrated with 50 mM Tris-HCl buffer (containing 150 mM NaCl, 2 mM Tris (2-carboxyethyl) phosphine hydrochloride (TCEP), pH 7.5). The peak fractions comprising VEEV nsp2pro-2 protease were analyzed on reducing SDS-PAGE, pooled and concentrated to 1–5 mg/mL. The concentration of VEEV nsp2pro-2 was determined as 2.9 mg/mL by A = 280 method. The theoretical extinction coefficient and molecular weight were calculated based on the primary structure of the proteins by ProtParam tool of ExPASy [38]. Aliquots were flash-frozen with liquid nitrogen and stored at −80 °C until further use.

### 4.6. Expression and Purification of the Substrates

The procedure of recombinant substrate expression and purification described by Bozóki et al. [23,24] was optimized to a small-scale to support microplate-based specificity screening measurements. In this setup, the following parameters were changed compared to the original protocol: (i) 2.5 mL starter culture was added to 15 mL LB containing 100 µg/mL ampicillin in a 50-mL centrifuge tube; (ii) cells were grown at OD_600_ = 0.5–0.6; (iii) after IPTG induction, cells were incubated for 4 h at 37 °C; (iv) the volume of the lysis buffer was reduced to 1 mL.

His_6_-MBP-mEYFP substrates representing the wild-type, P4-Glu, P4-Thr, P4-Arg, P4-Gly, P1-Gly, P1′-Thr, and P2′-Ser variants of SFV nsP12 cleavage site were expressed and purified based on the previously described protocol. After the induction of expression by IPTG, the incubation was followed by addition of tetracycline to the cell suspensions (200 µg/mL final concentration) to arrest protein translation and enable improved folding of protein substrates [24]. After the treatment cell were incubated for 2 h at 37°C, followed by harvesting the cells.

### 4.7. Gel Electrophoresis

Wild-type His_6_-MBP-EYHAGA↓GVVETP-mEYFP substrates attached to Ni-NTA magnetic agarose beads (SAMBs) were suspended in elution buffer (50 mM NaH_2_PO_4_, 300 mM NaCl, 500 mM imidazole, 0.05% Tween 20, pH 8.0) or cleavage buffer (50 mM NaH_2_PO_4_, 300 mM NaCl, 0.05% Tween 20, pH 7.5). SAMBs in cleavage buffer were digested by TEV PR (at 7.1 µM final concentration) and VEEV nsP2pro-2 (at 5.2 µM final concentration) on 30 °C overnight. TEV (S219V) PR stock solution was purified according to Kapust et al. [28].

For native PAGE analysis, samples were prepared from 10 µL of each reaction with 2× loading buffer (62.5 mM Tris-HCI, pH 6.8, 25% glycerol, 0.01% bromophenol Blue). After electrophoresis, the gels were illuminated by using Dark Reader Blue Transilluminator (Labgene Scientific, Châtel-Saint-Denis, Switzerland), and subsequently proteins were detected by PageBlue Protein Staining Solution (Thermo Fisher Scientific, Waltham, MA, USA).

For reducing SDS-PAGE, samples were supplemented with 6x loading buffer (300 mM Tris, pH 6.8, 20% glycerol, 0.05% bromophenol blue, 12% SDS, 100 mM β-mercaptoethanol), denatured at 95 °C for 10 min, followed by electrophoresis. Proteins were detected in the gels by PageBlue Protein Staining Solution (Thermo Fisher Scientific, Waltham, MA, USA).

### 4.8. Microplate-Based Specificity Studies of VEEV nsp2pro-2

The specificity of VEEV nsP2pro-2 was studied by recombinant substrates containing the cleavage site sequences listed in Table 1a. We applied a 96-well microplate-based adaptation of the Ni-NTA magnetic bead-based assay platform described previously by Bozóki et al. [24]. For cleavage reactions, VEEV nsP2pro-2 (2.1–6.0 µM final concentration) was incubated with His_6_-MBP-mEYFP substrates (1–5 µM final concentration) at 30 °C while continuously shaking at 600 rpm. Incubation time was 40 min in the case of P5, P4, P1, and P2′ variants, while it was 20 h for P1′-modified substrates. The pH of the applied buffers was 7.5. Flat-bottom 96-well plates were applied, using 96-Well Magnet Type A magnetic particle concentrator (Qiagen, Hilden, Germany). The final volume of the assay samples was 70 µL per well. During incubation and washing of the beads, plates were shaken by a digital shaker (IKA MS3). The detection of the fluorescence the products and substrates were performed by using either Victor^2^ Wallac 1420 device with 544/15 nm excitation and 590/10 emission filters or Biotek Synergy H1 device at 510 nm excitation and 540 nm emission wavelengths.

Statistical analysis of differences was performed by GraphPad unpaired *t*-test (https://www.graphpad.com/quickcalcs/ttest1/). With the exception of P1′ variants, one-way analysis of variances (ANOVA) was also applied. In the case of an unpaired t-test, *p*-value < 0.05 was considered to be statistically significant, while in the case of one-way ANOVA, the null hypothesis (H_0_), namely that the means of the tested populations (μ_1_ and μ_2_) are equal (H_0_: μ_1_ = μ_2_), was rejected if F > F_critical._

### 4.9. Kinetic Studies of VEEV nsP2pro-2

Kinetic measurements were carried out using the microtube-based Ni-NTA magnetic bead-based assay platform developed previously by Bozóki et al. [24].

Time-course measurements were performed on His_6_-MBP-EYHAGA↓GVVETP-mEYFP substrate (at 0.02 mM final concentration) processed by VEEV nsP2pro-2 (at 3.0 µM final total concentration). The product formation was followed by measuring fluorescence after for 0, 5, 10, 15, 20, 30, 50, 70, and 90 min at 30 °C, while continuously shaking at 600 rpm. Molar concentration of the C-terminal fluorescent cleavage product in the reaction samples were plotted against time (min). Linear regression was performed, and the parameters of the fitted lines were determined by GraphPad Prism version 5.00.

Substrate-dependent kinetic studies were performed on substrates at VEEVnsP2pro-2 different enzyme concentrations listed in Table 2. The samples were incubated for 10 min at 30 °C, while continuously shaking at 600 rpm. Kinetic parameters were determined at <20% substrate turnover by Michaelis–Menten non-linear regression analysis using GraphPad Prism version 5.00 for Windows (GraphPad Software, La Jolla, CA, USA, www.graphpad.com). The standard deviation values for the k_cat_/K_M_ vales were calculated based on Boross et al. [39]. Significance of the differences between the k_cat_/K_M_ values of the tested variants and the wild-type substrate were determined by GraphPad unpaired *t*-test (https://www.graphpad.com/quickcalcs/ttest1/). *p*-value < 0.05 was considered to be statistically significant.

### 4.10. In Silico Structural Analyses

The structural analyses were performed based on a modeled complex of VEEV nsP2 protease and LQEAGA↓GSVETP substrate [5]; the coordinate file was kindly provided by Patricia M. Legler (Center for Bio/molecular Science and Engineering, U.S. Naval Research Laboratory).

The sequence of the original model and the experimentally examined protein was aligned with Clustal Omega [40]. Both the model protein and substrate found in the crystal structure were mutated to reproduce the in vitro structures, by applying the FoldX software [41]. To the N-termini of both molecules, an N-acetyl group was connected, while a methylamide group was added to the C-termini. The protonation states were attributed by the Chimera software [42], the active site contained a deprotonated cysteine and a protonated histidine residue. Prior to the simulations, the systems were immersed in water. For the simulations, the Amber16 software [43] was used. The protein and the substrate were parametrized by the FF14SB force field [44], and the surrounding water molecules were described by the TIP3P model [45]. For the simulations, the system was first minimized via the steepest descent method for 5000 steps, followed by the conjugate gradient method for a further 5000 steps. The system was gradually heated to 300 K during a 2-ns-long simulation, and equilibrated for 2 ns at 300 K, applying 1-fs timesteps. For temperature regulation, the Langevin dynamics method was used. For bonds involving hydrogen atoms, SHAKE was performed. The system was further heated to 400 K during a 0.5-ns-long simulation, and equilibrated for 0.5 ns at 400 K. A simulated annealing cycle was applied to the system, cooling it to 5 K during a 1-ns-long simulation, followed by heating to 400 K during 0.5 ns, and equilibrating at that temperature for 0.5 ns, under NPT conditions. The cycle was repeated two times, heating the system to 300 K in the second cycle. A further 0.5-ns-long equilibration was run at 300 K. During the simulations, harmonic restraints of 50 kcal mol^−1^ Å^−2^ were applied between the sulfur atom of the catalytic cysteine and the substrate carbonyl oxygen of the Ala residue at the cleavage site, and also between the donor nitrogen atom of the catalytic histidine and the backbone nitrogen of the Gly residue at the cleavage site. Convergence during the last equilibration simulation was monitored with RMSD calculations, performed by the cpptraj [46] program of Amber16, with the average of coordinates over the equilibration trajectory used as reference.

DynaMut web server was applied to prepare mutant complexes and predict energy changes upon point mutations of substrate residues, the ≥0 kcal/mol change in folding free energy (ΔΔG) was considered to be stabilizing, while ΔΔG < 0 was viewed as destabilizing [47]. Hydropathy plots were prepared for the cleavage site sequences (P5–P5′) using the ProtScale module of ExPASy web server (https://web.expasy.org/protscale) [38], setting a 3-residue window for calculation and using the Kyte and Doolittle hydropathicity scale [48]. Residue volumes were retrieved from the literature [49]. LigPlot+_v2.1 program was used for 2D visualization of interaction [50]. Structural figures were prepared by using PyMOL Molecular Graphics System (Version 1.3 Schrödinger, LLC).

## Figures and Tables

**Table:** (**a**)

SFV nsP12 Cleavage Site	Cleavage Site Sequence	k_cat_ (s^−1^) (*10^−4^)	K_M_ (mM) (*10^−4^)	k_cat_/K_M_ (mM^−1^ s^−1^)	DynaMut ΔΔG (kcal/mol)
wild-type	EYHAGA↓GVVETP	3 ± 0.1	56 ± 9	0.06 ± 0.010	-
P5-Gln	E**Q**HAGA↓GVVETP	-	-	-	−0.222
P4-Glu	EY**E**AGA↓GVVETP	25 ± 0.7	83 ± 6	0.29 ± 0.024	0.175
P4-Thr	EY**T**AGA↓GVVETP	13 ± 0.06	60 ± 8	0.22 ± 0.032	−0.133
P4-Arg	EY**R**AGA↓GVVETP	8 ± 0.4	81 ± 10	0.10 ± 0.015	−0.200
P4-Gly	EY**G**AGA↓GVVETP	5 ± 0.3	91 ± 10	0.05 ± 0.008	−0.349
P2-Ala	EYHA**A**A↓GVVETP	-	-	-	−0.536
P2-Val	EYHA**V**A↓GVVETP	-	-	-	0.495
P1-Gly	EYHAG**G**↓GVVETP	5 ± 0.3	92 ± 10	0.05 ± 0.007	0.188
P1-Val	EYHAG**V**↓GVVETP	-	-	-	1.207
P1′-Thr *	EYHAGA↓**T**VVETP	0.6 ± 0.004	42 ± 8	0.02 ± 0.003	1.150
P1′-Val *	EYHAGA↓**V**VVETP	-	-	-	1.314
P1′-Pro *	EYHAGA↓**P**VVETP	-	-	-	0.875
P1′-His *	EYHAGA↓**H**VVETP	-	-	-	1.269
P1′-Cys *	EYHAGA↓**C**VVETP	-	-	-	1.718
P1′-Ser *	EYHAGA↓**S**VVETP	-	-	-	1.731
P1′-Arg *	EYHAGA↓**R**VVETP	-	-	-	1.166
P1′-Leu *	EYHAGA↓**L**VVETP	-	-	-	1.830
P1′-Lys *	EYHAGA↓**K**VVETP	-	-	-	1.336
P1′-Glu *	EYHAGA↓**E**VVETP	-	-	-	1.506
P1′-Tyr	EYHAGA↓**Y**VVETP	-	-	-	2.082
P1′-Ile	EYHAGA↓**I**VVETP	-	-	-	1.085
P1′-Gln	EYHAGA↓**Q**VVETP	-	-	-	1.050
P1′-Asn	EYHAGA↓**N**VVETP	-	-	-	1.962
P1′-Asp	EYHAGA↓**D**VVETP	-	-	-	0.971
P1′-Ala	EYHAGA↓**A**VVETP	-	-	-	1.107
P1′-Met	EYHAGA↓**M**VVETP	-	-	-	0.883
P1′-Trp	EYHAGA↓**W**VVETP	-	-	-	2.477
P1′-Phe	EYHAGA↓**F**VVETP	-	-	-	2.085
P2′-Pro	EYHAGA↓G**P**VETP	-	-	-	0.427
P2′-Ser	EYHAGA↓G**S**VETP	5 ± 0.2	81 ± 8	0.06 ± 0.006	−0.068

**Table:** (**b**)

Enzyme	Cleavage Site Sequence	Substrate	k_cat_ (s^−1^) (*10^−4^)	K_M_ (mM) (*10^−4^)	k_cat_/K_M_ (mM^−1^ s^−1^)
VEEV nsP2pro-1 *	DVEELEYHAGA↓GVVETP	Protein	33 ± 20	2300 ± 200	0.144 ± 0.01
	AETGVVDVDVEELEYHAGA↓GVVETP	Protein	500 ± 30	6000 ± 2000	0.083 ± 0.03
VEEV nsP2pro-1 **	EYHAGA↓GVVETP	Peptide	160 ± 10	5800 ± 900	0.028 ± 0.005

**Table 2:** The list of the substrates on which VEEV nsP2pro-2 kinetic studies were performed. The His_6_-MBP-mEYFP substrates are listed. The corresponding substrate and enzyme concentrations are indicated.

Substrate	Substrate Concentration (µM)	Applied VEEV nsP2pro-2 Concentration (µM)
wt	3.15–63.3	3.94
P4-Glu	0.71–28.5	1.46
P4-Thr	0.76–30.5	1.88
P4-Arg	0.84–21.1	3.75
P4-Gly	0.71–14.2	5.21
P1-Gly	0.79–15.8	5.21
P1′-Thr	1.06–21.1	7.29
P2′-Ser	0.76–19.4	4.11

## Supplementary Materials

Supplementary materials can be found at https://www.mdpi.com/1422-0067/21/20/7686/s1. Figure S1. Alignment of five alphavirus nsP2 sequences. Figure S2. Generation dsDNA inserts coding for SFV nsP12 P1′ variants by random mutagenesis. Figure S3. Root mean square deviation (RMSD) of enzyme–substrate complex during simulation. Table S1. Oligonucleotide primers used for the generation and sequencing of expression plasmids coding for VEEV nsP2 and nsP2pro-2. Table S2. List of the chemically synthetized oligonucleotide primers.

## Abbreviations

MBP	Maltose Binding Protein
mEYFP	Monomeric Enhanced Yellow Fluorescent Protein
nsP	Non-structural Protein
PR	Protease
SFV	Semliki forest virus
TEV	Tobacco Etch Virus
VEEV	Venezuelan Equine Encephalitis Virus
