# Supplementary material for: Specificity Studies of the Venezuelan Equine Encephalitis Virus Non-Structural Protein 2 Protease Using Recombinant Fluorescent Substrates
Source: Int J Mol Sci. 2020 Oct 16;21(20):7686. doi: 10.3390/ijms21207686 (PMC7593941; doi:10.3390/ijms21207686)
Supplement: Supplementary file 1 [file ijms-21-07686-s001.zip › Table S2.pdf]

Table S2. List of the chemically synthetized oligonucleotide primers. The primers that were applied for the generation of pDEST-His$_6$-MBP-mEYFP expression plasmids coding for the wild-type, P5, P4, P1, P2 and P1′ variants of SFV nsP12 (EYHAGA↓GVVETP) cleavage site sequence are listed. Modified cleavage site residues are bold and red. FWD=forward, REV =reverse.

Cleavage site sequence	Oligonucleotide primers
EYHAGA↓GVVETP	FWD:5′-TAAAGAATATCATGCTGGTGCTGGTGTTGTTGAAACACCGG-3′<br>REV:5′–CTAGCCGGTGTTTCAACAACACCAGCACCAGCATGATATTCTTTAAT-3′
EYHAGA↓**Y**VVETP	FWD:5′-TAAAGAATATCATGCTGGTGCTTATGTTGTTGAAACACCGG-3′<br>REV:5′–CTAGCCGGTGTTTCAACAACATAAGCACCAGCATGATATTCTTTAAT-3′
EYHAGA↓**I**VVETP	FWD:5′-TAAAGAATATCATGCTGGTGCTATTGTTGTTGAAACACCGG-3′<br>REV:5′–CTAGCCGGTGTTTCAACAACAATAGCACCAGCATGATATTCTTTAAT-3′
EYHAGA↓**Q**VVETP	FWD:5′-TAAAGAATATCATGCTGGTGCTCAGGTTGTTGAAACACCGG-3′<br>REV:5′–CTAGCCGGTGTTTCAACAACCTGAGCACCAGCATGATATTCTTTAAT-3′
EYHAGA↓**N**VVETP	FWD:5′-TAAAGAATATCATGCTGGTGCTAACGTTGTTGAAACACCGG-3′<br>REV:5′–CTAGCCGGTGTTTCAACAACGTTAGCACCAGCATGATATTCTTTAAT-3′
EYHAGA↓**D**VVETP	FWD:5′-TAAAGAATATCATGCTGGTGCTGATGTTGTTGAAACACCGG-3′<br>REV:5′–CTAGCCGGTGTTTCAACAACATCAGCACCAGCATGATATTCTTTAAT-3′
EYHAGA↓**A**VVETP	FWD:5′-TAAAGAATATCATGCTGGTGCTGCGGTTGTTGAAACACCGG-3′<br>REV:5′–CTAGCCGGTGTTTCAACAACCGCAGCACCAGCATGATATTCTTTAAT-3′
EYHAGA↓**M**VVETP	FWD:5′-TAAAGAATATCATGCTGGTGCTATGGTTGTTGAAACACCGG-3′<br>REV:5′–CTAGCCGGTGTTTCAACAACCATAGCACCAGCATGATATTCTTTAAT-3′
EYHAGA↓**W**VVETP	FWD:5′-TAAAGAATATCATGCTGGTGCTTGGGTTGTTGAAACACCGG-3′<br>REV:5′–CTAGCCGGTGTTTCAACAACCCAAGCACCAGCATGATATTCTTTAAT-3′
EYHAGA↓**F**VVETP	FWD:5′-TAAAGAATATCATGCTGGTGCTTTTGTTGTTGAAACACCGG-3′<br>REV:5′–CTAGCCGGTGTTTCAACAACAAAAGCACCAGCATGATATTCTTTAAT-3′
E**Q**HAGA↓GVVETP	FWD:5′-TAAAGAACAGCATGCTGGTGCTGGTGTTGTTGAAACACCGG-3′<br>REV:5′–CTAGCCGGTGTTTCAACAACACCAGCACCAGCATGCTGTTCTTTAAT-3′
EY**E**AGA↓GVVETP	FWD:5′-TAAAGAATATGAAGCTGGTGCTGGTGTTGTTGAAACACCGG-3′<br>REV:5′–CTAGCCGGTGTTTCAACAACACCAGCACCAGCTTCATATTCTTTAAT-3′
EY**T**AGA↓GVVETP	FWD:5′-TAAAGAATATACCGCTGGTGCTGGTGTTGTTGAAACACCGG-3′<br>REV:5′–CTAGCCGGTGTTTCAACAACACCAGCACCAGCGGTATATTCTTTAAT-3′
EY**R**AGA↓GVVETP	FWD:5′-TAAAGAATATCGCGCTGGTGCTGGTGTTGTTGAAACACCGG-3′<br>REV:5′–CTAGCCGGTGTTTCAACAACACCAGCACCAGCGCGATATTCTTTAAT-3′
EY**G**AGA↓GVVETP	FWD:5′-TAAAGAATATGGTGCTGGTGCTGGTGTTGTTGAAACACCGG-3′<br>REV:5′–CTAGCCGGTGTTTCAACAACACCAGCACCAGCACCATATTCTTTAAT-3′
EYHA**A**A↓GVVETP	FWD:5′-TAAAGAATATCATGCTGCTGCTGGTGTTGTTGAAACACCGG-3′<br>REV:5′–CTAGCCGGTGTTTCAACAACACCAGCAGCAGCATGATATTCTTTAAT-3′
EYHA**V**A↓GVVETP	FWD:5′-TAAAGAATATCATGCTGTGGCTGGTGTTGTTGAAACACCGG-3′<br>REV:5′–CTAGCCGGTGTTTCAACAACACCAGCCACAGCATGATATTCTTTAAT-3′
EYHAG**G**↓GVVETP	FWD:5′-TAAAGAATATCATGCTGGTGGTGGTGTTGTTGAAACACCGG-3′<br>REV:5′–CTAGCCGGTGTTTCAACAACACCACCACCAGCATGATATTCTTTAAT-3′
EYHAG**V**↓GVVETP	FWD:5′-TAAAGAATATCATGCTGGTGTTGGTGTTGTTGAAACACCGG-3′<br>REV:5′–CTAGCCGGTGTTTCAACAACACCAACACCAGCATGATATTCTTTAAT-3′
EYHAGA↓G**P**VETP	FWD:5′-TAAAGAATATCATGCTGGTGCTGGTCCGGTTGAAACACCGG-3′<br>REV:5′–CTAGCCGGTGTTTCAACCGGACCAGCACCAGCATGATATTCTTTAAT-3′
EYHAGA↓G**S**VETP	FWD:5′-TAAAGAATATCATGCTGGTGCTGGTAGCGTTGAAACACCGG-3′<br>REV:5′–CTAGCCGGTGTTTCAACCGTACCAGCACCAGCATGATATTCTTTAAT-3′
