# Supplementary material for: Specificity Studies of the Venezuelan Equine Encephalitis Virus Non-Structural Protein 2 Protease Using Recombinant Fluorescent Substrates
Source: Int J Mol Sci. 2020 Oct 16;21(20):7686. doi: 10.3390/ijms21207686 (PMC7593941; doi:10.3390/ijms21207686)
Supplement: Supplementary file 1 [file ijms-21-07686-s001.zip › Table S1.pdf]

Table S1. Oligonucleotide primers used for the generation and sequencing of expression plasmids coding for VEEV nsP2 and nsP2pro-2. PE2285, PE2286, PE2287, and PE2288 primers were used to sequence the C-terminal, while PE2730 and PE2731 were applied to sequence the N-terminal domain of VEEV nsP2. Primer N2, N1, and C are referring to the nomenclature used by Tropea *et al.* [**27**]. FWD =forward, REV=reverse. Primer used for sequencing are marked with asterisks.

Code	Primer Description	Sequence
PE2685	N1 - VEEV nsP2	5′-GGCTCGGAGAACCTGTACTTCCAGGGCTCAGTGGA-GACACCTCGTG-3′
PE2687	N1 - VEEV nsp2pro-2	5′-GGCTCGGAGAACCTGTACTTCCAGGCCAAGTACCCTGGGAATTTCACT-3′
PE2686	C	5′-GGGGACCACTTTGTACAAGAAAGCTGGGTTATTAT-GTATAAATGTTGGTCAAGGTTGATGAAAGC-3′
PE277	N2	5′-GGGGACAAGTTTGTACAAAAAAGCAGGCTCGGAGA-ACCTGTACTTCCAG-3′
PE2285	FWD*	5′- ATGCGTGAGGTTCTTTGGAC-3′
PE2286	FWD*	5′-CAGCAGTGTGAAGACCATGC-3′
PE2287	REV*	5′-AGGACAGTTCTGCCCTTCAA-3′
PE2288	REV*	5′-TTGGTCAAGGTTGATGAAAGC-3′
PE2730	FWD*	5′-CCTTCCATGAATTCGCCTAC-3′
PE2731	FWD*	5′-AACGACGAATCCGAAAGAGA-3′
