# Supplementary material for: Specificity Studies of the Venezuelan Equine Encephalitis Virus Non-Structural Protein 2 Protease Using Recombinant Fluorescent Substrates
Source: Int J Mol Sci. 2020 Oct 16;21(20):7686. doi: 10.3390/ijms21207686 (PMC7593941; doi:10.3390/ijms21207686)
Supplement: Supplementary file 1 [file ijms-21-07686-s001.zip › Figure S1.pdf]

Figure S1. Alignment of five alphavirus nsP2 sequences. Sequence of VEEV nsP2pro-2 is bold, while sequence of VEEV nsP2pro-1 is underlined. Residues of 436-457 positions are marked with yellow. Some positions of VEEV sequence that are mentioned in the main text are labeled.

```
VEEV         GSVETPRGLIKVTSYAGEDKIGSYAVLSPQAVLKSEKLSCIHPLAEQVIVITHSGRKGRYAVEPYHGKVVVPEGHAIPVQDFQALSESATIVYNEREFVNRYLHHIATHGGALNTDEEYY  120
Sindbis      ALVETPRGHVRIIPQANDRMIGQYIVVSPNSVLKNAKLAPAHPLADQVKIITHSGRSGRYAVEPYDAKVLMPAGGAVPWPEFLALSESATLVYNEREFVNRKLYHIAMHGPAKNTEEEQY  120
Chikungunya  GIIETPRGAIKVTAQPTDHVVGEYLVLSPQTVLRSQKLSLIHALAEQVKTCTHSGRVGRYAVEAYDGRVLVPSGYAISPEDFQSLSESATMVYNEREFVNRKLHHIAMHGPALNTDEESY  120
Sagiyama     GVVETPRNALKVTPQAHDHLIGSYLILSPQTVLKSEKLAPIHPLAEQVTVMTHSGRSGRYPVDKYDGRVLIPTGAAIPVSEFQALSESATMVYNEREFINRKLHHIALYGPALNTDEESY  120
Semliki      GVVETPRSALKVTAQPNDVLLGNYVVLSPQTVLKSSKLAPVHPLAEQVKIITHNGRAGRYQVDGYDGRVLLPCGSAIPVPEFQALSESATMVYNEREFVNRKLYHIAVHGPSLNTDEENY  120
             . :****. :::     :  :*.* ::**::**:. **:  * **:**   **.** *** *: *..:*::* * *:   :* :******:*******:** *:*** :* : **:** *

VEEV         KTVKPSEHDGEYLYDIDRKQCVKKELVTGLGLTGELVDPPFHEFAYESLRTRPAAPYQVPTIGVYGVPGSGKSGIIKSAVTKKDLVVSAKKENCAEIIRDVKKMKGLDVNARTVDSVLLN  240
Sindbis      KVTKAELAETEYVFDVDKKRCVKKEEASGLVLSGELTNPPYHELALEGLKTRPAVPYKVETIGVIGTPGSGKSAIIKSTVTARDLVTSGKKENCREIEADVLRLRGMQITSKTVDSVMLN  240
Chikungunya  ELVRAERTEHEYVYDVDQRRCCKKEEAAGLVLVGDLTNPPYHEFAYEGLKIRPACPYKIAVIGVFGVPGSGKSAIIKNLVTRQDLVTSGKKENCQEITTDVMRQRGLEISARTVDSLLLN  240
Sagiyama     EKVRAERAETEYVFDVDKKACIKKEEASGLVLTGDLINPPFHEFAYEGLKIRPAAPYHTTIIGVFGVPGSGKSAIIKNMVTTRDLVASGKKENCQEIMNDVKRQRGLDVTARTVDSILLN  240
Semliki      EKVRAERTDAEYVFDVDKKCCIKREEASGLVLVGELTNPPFHEFAYEGLKIRPSAPYKTTVVGVFGVPGSGKSAIIKSLVTKHDLVTSGKKENCQEIVNDVKKHRGLDIQAKTVDSILLN  240
             : .: .  : **::*:*:: * *:* .:** * *:* :**:**:* *.*: **: **:    :** *.******.***. ** :***.*.***** **  ** : :*::: ::****::**

VEEV         GCKHPVETLYIDEAFACHAGTLRALIAIIRPK-KAVLCGDPKQCGFFNMMCLKVHFNH---EICTQVFHKSISRRCTKSVTSVVSTLFYDKKMRTTNPKETKIVIDTTGSTKPKQDDLIL  356
Sindbis      GCHKAVEVLYVDEAFACHAGALLALIAIVRPRKKVVLCGDPMQCGFFNMMQLKVHFNHPEKDICTKTFYKYISRRCTQPVTAIVSTLHYDGKMKTTNPCKKNIEIDITGATKPKPGDIIL  360
Chikungunya  GCNRPVDVLYVDEAFACHSGTLLALIALVRPRQKVVLCGDPKQCGFFNMMQMKVNYNH---NICTQVYHKSISRRCTLPVTAIVSSLHYEGKMRTTNEYNKPIVVDTTGSTKPDPGDLVL  357
Sagiyama     GCKRGVENLYVDEAFACHSGTLLALIALVRPSGKVVLCGDPKQCGFFNLMQLKVHYNH---NICTRVLHKSISRRCTLPVTAIVSTLHYQGKMRTTNRCNTPIQIDTTGSSKPASGDIVL  357
Semliki      GCRRAVDILYVDEAFACHSGTLLALIALVKPRSKVVLCGDPKQCGFFNMMQLKVNFNH---NICTEVCHKSISRRCTRPVTAIVSTLHYGGKMRTTNPCNKPIIIDTTGQTKPKPGDIVL  357
             **.: *: **:*******:*:* ****:::*  *.****** ******:* :**::**   :***.. :* ******  **::**:*.*  **:***  :. * :* ** :**  .*::*
                                                                                                   436                  457                475
VEEV         TCFRGWVKQLQIDYKGNEIMTAAASQGLTRKGVYAVRYKVNENPLYAPTSEHVNVLLTRTEDRIVWKTLAGDPWIKTLTAKYPGNFTATIEEWQAEHDAIMRHILERPDPTDVFQNKANV  476
Sindbis      TCFRGWVKQLQIDYPGHEVMTAAASQGLTRKGVYAVRQKVNENPLYAITSEHVNVLLTRTEDRLVWKTLQGDPWIKQPTNIPKGNFQATIEDWEAEHKGIIAAINSPTPRANPFSCKTNV  480
Chikungunya  TCFRGWVKQLQIDYRGYEVMTAAASQGLTRKGVYAVRQKVNENPLYASTSEHVNVLLTRTEGKLVWKTLSGDPWIKTLQNPPKGNFKATIKEWEVEHASIMAGICSHQMTFDTFQNKANV  477
Sagiyama     TCFRGWVKQLQIDYRGHEVMTAAASQGLTRKGVYAVRQKVNENPLYSPLSEHVNVLLTRTENRLVWKTLSGDPWIKVLTNVPRGDFSATLEEWQEEHDGIMRVLNERPAEVDPFQNKAKV  477
Semliki      TCFRGWVKQLQLDYRGHEVMTAAASQGLTRKGVYAVRQKVNENPLYAPASEHVNVLLTRTEDRLVWKTLAGDPWIKVLSNIPQGNFTATLEEWQEEHDKIMKVIEGPAAPVDAFQNKANV  477
             ***********:** * *:***************** ********:  ************.::***** ******       *:* **:::*: **  *:  :          : *. *::*
             477                                 509                            546
VEEV         CWAKALVPVLKTAGIDMTTEQWNTVD-YFETDKAHSAEIVLNQLCVRFFGLDLDSGLFSAPTVPLSIR-------NNHWDNSPSPNMYGLNKEVVRQLSRRYPQLPRAVATGRVYDMNTG  588
Sindbis      CWAKALEPILATAGIVLTGCQWSELFPQFADDKPHSAIYALDVICIKFFGMDLTSGLFSKQSIPLTYHPADSARPVAHWDNSPGTRKYGYDHAIAAELSRRFPVFQLA-GKGTQLDLQTG  599
Chikungunya  CWAKSLVPILETAGIKLNDRQWSQIIQAFKEDKAYSPEVALNEICTRMYGVDLDSGLFSKPLVSVYYA-------DNHWDNRPGGKMFGFNPEAASILERKYPFTKGKWNINKQICVTTR  590
Sagiyama     CWAKCLVQVLETAGIRMTADEWNTIL-AFREDRAYSPEVALNEICTRYYGVDLDSGLFSAQSVSLFYE-------NNHWDNRPGGRMYGFNHEVARKYAARFPFLRGNMNSGLQLNVPER  589
Semliki      CWAKSLVPVLDTAGIRLTAEEWSTIITAFKEDRAYSPEVALNEICTKYYGVDLDSGLFSAPKVSLYYE-------NNHWDNRPGGRMYGFNAATAARLEARHTFLKGQWHTGKQAVIAER  590
             ****.*  :* **** :.  :*. :    *  *: :*    .*: :* : :*:** *****   : :               **** *. . :* :   .     :.         .    :
                                                                                                                               698 702
VEEV         TLRNYDPRINLVPVNRRLPHALVLHHNEHPQSDFSSFVSKLKGRTVLVV-GEKLSVPGKMVDWLSDRP--EATFRARLDLGIPGDVPKYDIIFVNVRTPYKYHHYQQCEDHAIKLSMLTK  705
Sindbis      RTRVISAQHNLVPVNRNLPHALVPEYKEKQPGPVKKFLNQFKHHSVLVVSEEKIEAPRKRIEWIAPIGIAGADKNYNLAFGFPPQ-ARYDLVFINIGTKYRNHHFQQCEDHAATLKTLSR  718
Chikungunya  RIEDFNPTTNIIPANRRLPHSLVAEHRPVKGERMEWLVNKINGHHVLLVSGYNLVLPTKRVTWVAPLGVRGADYTYNLELGLPATLGRYDLVVINIHTPFRIHHYQQCVDHAMKLQMLGG  710
Sagiyama     KLQPFSAECNIVPSNRRLPHALVTSYQQCRGERVEWLLKKIPGHQMLLVSEYNLAIPHKRVFWIAPPRVSGADRTYDLDLGLPMDAGRYDLVFVNIHTEYRQHHYQQCVDHSMRLQMLGG  709
Semliki      KIQPLSVLDNVIPINRRLPHALVAEYKTVKGSRVEWLVNKVRGYHVLLVSEYNLALPRRRVTWLSPLNVTGADRCYDLSLGLPADAGRYDLVFVNIHTEFRIHHYQQCVDHAMKLQMLGG  710
               .  .   *::* **.***:**  :.      .. ::.:.     :*:*    ::  * : : *::        *      * :*:*    :**::.:*: * :: **:*** **:  *. *
                                                                                                   785
VEEV         KACLHLNPGGTCVSIGYGYADRASESIIGAIARQFKFSRVCKPKSSLEETEVLFVFIGYDRKA-RTHNPYKLSSTLTNIYTGSRLHEAGC          794
Sindbis      SALNCLNPGGTLVVKSYGYADRNSEDVVTALARKFVRVSAARPDCVSSNTEMYLIFRQLDNSRTRQFTPHHLNCVISSVYEGTRDGVGA-          807
Chikungunya  DSLRLLKPGGSLLIRAYGYADRTSERVICVLGRKFRSSRALKPPCVTSNTEMFFLFSNFDNGR-RNFTTHVMNNQLNAAFVGQAT-RAGC          798
Sagiyama     DSLHLLRPGGSLLMRAYGYADRVSEMVVTALARKFSAFRVLRPACVTSNTEVFLLFSNFDNGR-RAVTLHQANQKLSSMYACNGLHTAGC          798
Semliki      DALRLLKPGGNLLMRAYGYADKISEAVVSSLSRKFSSARVLRPDCVTSNTEVFLLFSNFDNGK-RPSTLHQMNTKLSAVYAGEAMHTAGC          799
             .:   *.***. :  .*****: ** ::  :.*:*     . :* .  .:**: ::*    *.    *  . :  .  :.  :       ..
```
