# Supplementary material for: Specificity Studies of the Venezuelan Equine Encephalitis Virus Non-Structural Protein 2 Protease Using Recombinant Fluorescent Substrates
Source: Int J Mol Sci. 2020 Oct 16;21(20):7686. doi: 10.3390/ijms21207686 (PMC7593941; doi:10.3390/ijms21207686)

Figure S2. Generation dsDNA inserts coding for SFV nsP12 P1′ variants by random mutagenesis. Cloning steps are numbered from 1 to 5. The first step of cloning was the hybridization of complementary, *E. coli*-optimized oligonucleotide primers that coded for the SFV nsP12 cleavage site sequence (red). The primers were flanked by BamHI and NheI ‘sticky’ ends (green and blue, respectively), and contained a PacI restriction endonuclease recognition site prior to the coding sequence of cleavage site (purple) (**1**). After linearization of pT7-Blue-3 vector by BamHI and NheI restriction endonucleases (**2**), the annealed oligonucleotide primer pair was inserted into pT7-Blue-3 plasmid by ligation (**3**). Point mutations were introduced to the cleavage sites by random mutagenesis using an oligonucleotide primer degenerated at the codon corresponding to SFV nsP12 P1′ residue (yellow) (**4**). The short dsDNAs coding for different SFV nsP12 P1′ variants were released from the pT7-Blue-3 plasmid by digestion using PacI and NheI restriction enzymes (**5**), followed by cloning of the short dsDNAs into pDEST-His$_6$-MBP-mEYFP plasmids.

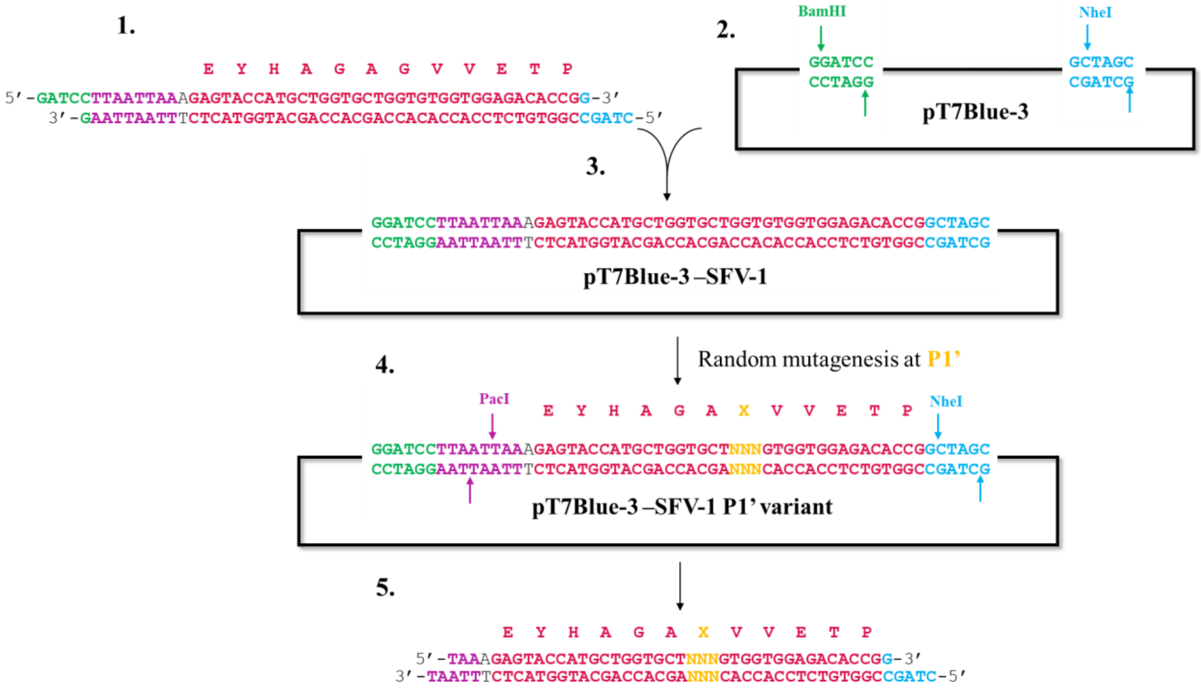

Supplement: Supplementary file 1 [file ijms-21-07686-s001.zip › Figure S2.pdf]
