# Supplementary material for: Specificity Studies of the Venezuelan Equine Encephalitis Virus Non-Structural Protein 2 Protease Using Recombinant Fluorescent Substrates
Source: Int J Mol Sci. 2020 Oct 16;21(20):7686. doi: 10.3390/ijms21207686 (PMC7593941; doi:10.3390/ijms21207686)

Figure S3. Root mean square deviation (RMSD) of enzyme-substrate complex during simulation. The plot shows RMSD as function of simulation time for the system with a deprotonated cysteine and a protonated histidine active site residues, average of coordinates used as reference.

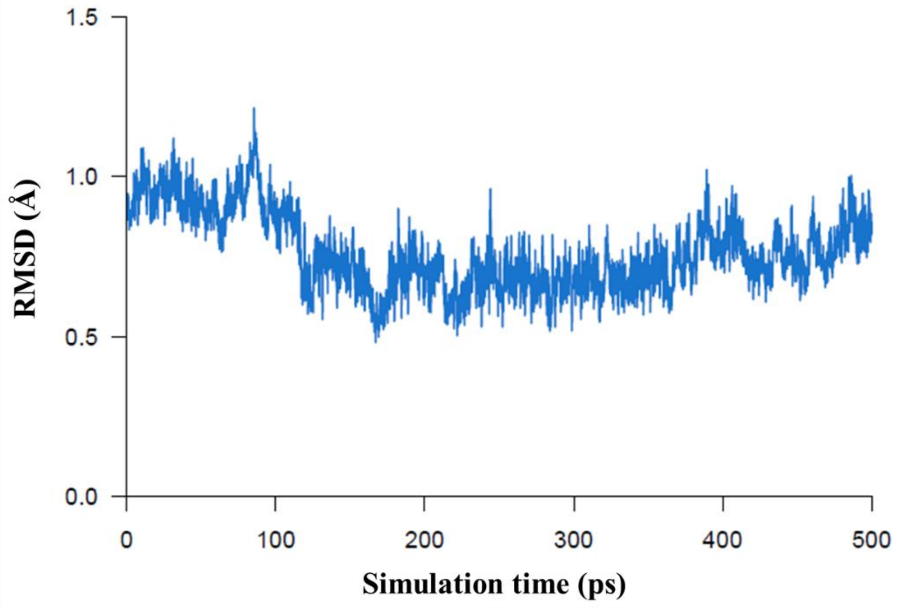

Supplement: Supplementary file 1 [file ijms-21-07686-s001.zip › Figure S3.pdf]
